# Social and Structural Determinants of Household Support for ART Adherence in Low- and Middle-Income Countries: A Systematic Review 

**DOI:** 10.3390/ijerph17113808

**Published:** 2020-05-27

**Authors:** Linda Campbell, Caroline Masquillier, Estrelle Thunnissen, Esther Ariyo, Hanani Tabana, Neo Sematlane, Anton Delport, Lorraine Tanyaradzwa Dube, Lucia Knight, Tair Kasztan Flechner, Edwin Wouters

**Affiliations:** 1 Centre for Population, Family and Health, University of Antwerp, 2000 Antwerp, Belgium; caroline.masquillier@uantwerpen.be (C.M.); estrelle.thunnissen@uantwerpen.be (E.T.); esther.ariyo@uantwerpen.be (E.A.); tair.kasztan@uantwerpen.be (T.K.F.); edwin.wouters@uantwerpen.be (E.W.); 2 School of Public Health, University of the Western Cape, Bellville 7535, South Africa; htabana@uwc.ac.za (H.T.); 3879990@myuwc.ac.za (N.S.); adelport@uwc.ac.za (A.D.); ldube@uwc.ac.za (L.T.D.); lknight@uwc.ac.za (L.K.)

**Keywords:** HIV, ART, adherence, family, household, structural factors, social determinants, social support

## Abstract

Adherence to HIV antiretroviral therapy (ART) is a crucial factor in health outcomes for people living with HIV (PLWH). Interventions to support ART adherence are increasingly focused on the household as a source of social support. This review aims to examine the social and structural determinants of support for ART adherence within households and families in low- and middle-income countries (LMICs). The review methodology followed the recommendations of the Preferred Reporting Items for Systematic Reviews and Meta-Analysis (PRISMA) guidelines. Seven databases were searched for peer-reviewed literature. The terms searched thematically covered (1) ART adherence, (2) household and family and (3) support and care. Thirty-three studies conducted in 15 LMICs were selected and a mixed methods synthesis was undertaken. Social and structural determinants affected the type, quality and amount of support for PLWH of all ages, which affected PLWH’s ART adherence. Gender norms affected the type of support that household members give to PLWH. Education moderated household support for ART adherence through literacy and language skills. Cultural context, religious beliefs, and social norms reinforced or undermined household support for ART adherence. Stigma affected disclosure, generated secrecy around giving medication and impeded access to support from the community. Supporting PLWH exacerbated economic hardship for household members. Health system dysfunction negatively impacted trust and communication between household members and health professionals. Intersecting social and structural determinants particularly affected the care given by household members who were older, female, with little education and low socioeconomic status. Household members were able to overcome some of these barriers when they received support themselves. Household interventions to support PLWH’s ART adherence should take structural factors into account to have maximum impact.

## 1. Introduction

The introduction of antiretroviral therapy (ART) has altered the course of the HIV pandemic, and transformed an infection with a high number of fatalities into a chronic, manageable disease [1]. The global roll-out of ART has seen reductions in morbidity and mortality of people living with HIV (PLWH) as well as in the onward transmission of the virus [2,3,4,5]. Since recommendations regarding the initiation of ART changed to a ‘treat-all’ approach, ART is now initiated as early as possible following diagnosis [6]. Access and adherence, however, remain key issues: in 2018, there were an estimated 37.9 million people living with HIV, most of whom live in low- and middle-income countries (LMICs), and in particular, sub-Saharan Africa, and yet only an estimated 62% of PLWH globally obtained ART [7,8]. While access to ART has improved in recent years and the number of people living with HIV (PLWH) accessing ART worldwide reached an unprecedented 24.5 million in June 2019, coverage for LMICs varies widely, from 9% (7%–13%) in Madagascar to 92% in Namibia (84%–>95%) [7,8]. Access to second-line and third-line regimens remains subject to cost and availability [9]. Furthermore, consistent, life-long ART adherence is vital for improving health outcomes and halting drug resistance [10,11,12,13,14,15]. Consequently, supporting PLWH to adhere optimally to ART is imperative, and effective public health interventions play a key role in supporting successful adherence [16,17,18,19]. 

To date, a variety of ART adherence interventions have used an individual-level or community-based approach in order to improve ART adherence rates, which have varied in impact [16,17,18,20,21]: examples include individual/group counselling, mHealth interventions such as SMS or telephone reminders, home visits from community health workers, outreach to connect PLWH with facilities, nutrition support, community-based social network support, pharmacist counselling, facility-based interventions and task shifting, e.g., from hospitals to clinics. A growing body of evidence drawing on socio-ecological theory emphasises the influence of social context on health-related behaviour as impacting intervention success [22,23,24,25]. For example, Wouters et al. (2014) explained that peer adherence support interventions impacted patient outcomes differently depending on the patient’s family or household context [26]. Increasing attention has therefore focused on the dynamics of the household, a micro-environment subsumed within the interpersonal network level of socio-ecological models [24,25,27]. It is important to recognise the kind of support that households either offer or withhold from PLWH [27,28], including instrumental, emotional and informational support [29]; for example, reminding PLWH to take medication or accompanying them to clinic visits, giving emotional support and listening to worries and concerns, and sharing responsibility for preventing onward transmission. Masquillier et al. (2015) posited that when household support for PLWH is characterised by behaviours supporting disclosure, discussion and prevention, the household is deemed to be “HIV competent”: household HIV competency creates a health-enabling environment for PLWH and has positive effects on ART adherence and prevention behaviour [20]. 

**Figure 1:**
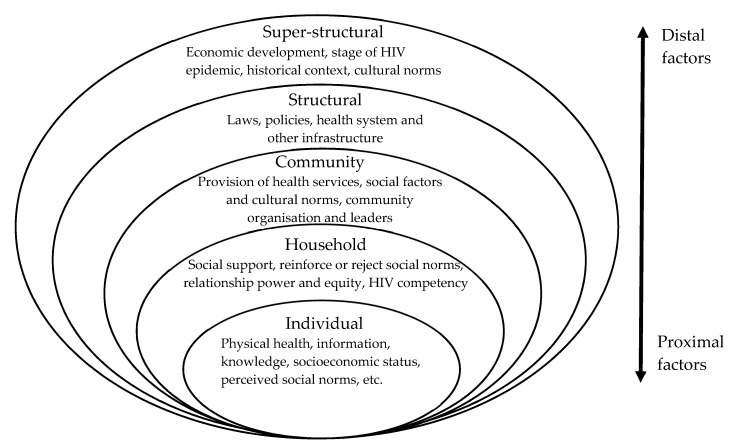
Adapted socio-ecological model of social and structural determinants of HIV transmission, vulnerability and antiretroviral therapy (ART) adherence, drawing together the frameworks proposed by Sweat and Denison (1995), Baral et al. (2013), and Wouters (2012) [24,28,31].

It is important to note that household HIV competency and support for ART adherence may be affected by social and structural factors [21]. Social and structural factors are broadly conceptualised as the economic, social, political and policy factors that affect an individual’s vulnerability to acquiring HIV or ability to manage life with HIV, and vary according to location, culture, and resource availability [24,30]. These factors are located on a continuum from distal factors at the macro level (far from the individual) to proximal factors at the micro level (close to the individual) (see Figure 1) [24,31]. The underlying assumption is that causal pathways link social and structural factors with the risk of HIV transmission and ART adherence [23] and that a distal, structural factor such as health system infrastructure can affect an individual’s risk of acquiring HIV or adhering to ART through pathways that cut through these levels. Individual risk is therefore not equal for everyone: societal social norms—cultural rules about behaviour reflecting societal values—are manifested in organisations and institutions at each of these levels [32], which determine to whom societal power is distributed [22]. Social inequalities create differing patterns of risk and vulnerability to acquiring HIV and adhering to ART, generating health inequalities between different populations [22,33]. Such inequalities are apparent at the household level, with household members possessing different levels of power and agency [23]. Intersectionality theory posits that PLWH experience a variety of different social positions based on race, gender, (dis)ability, etc., and therefore experience compounded vulnerability depending on their different levels of social power [34]. Examining the social and structural determinants that affect household support for ART adherence is therefore key to developing effective household interventions for ART adherence. 

Previous reviews in LMICs and high-income countries (HICs) have examined social and structural factors affecting adherence to ART at the patient level [35,36,37,38,39,40,41,42,43,44,45]. However, most studies regarding family and households of PLWH have focused upon relational factors such as family dynamics and family structure [46] instead of social determinants, or have mentioned a positive association between social support and better adherence rates [45,47,48]. Four reviews on determinants of ART adherence in PLWH in LMICs have briefly mentioned social and structural factors affecting family or household members [35,38,40,43]; Vreeman (2008), for example, mentions that low parental education is associated with non-adherence in children [43]. These reviews have not, however, examined the mechanisms by which social and structural factors—such as education—impact family or household support for ART adherence, and do not examine whether resulting differences in type, amount or quality of household support impacts ART adherence of PLWH. Additionally, none have examined whether there is a combined impact of intersecting social and structural factors on household member support for ART adherence. This review, therefore, focused on findings that elaborated on or complemented previous results in order to avoid repetition. 

**Figure 2:**
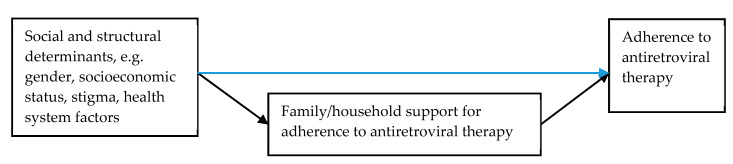
Social and structural determinants may directly affect the adherence behaviour of people living with HIV, or indirectly via the type and quality of support given by household members. This review concentrates on the indirect relationship.

This systematic literature review, therefore, aimed (1) to review the literature that examines social and structural determinants of household member support for ART adherence within households and families in LMICs; (2) to examine how social determinants affect household and family support for ART adherence (see Figure 2), including the type, duration and quality of support given to PLWH; and (3) to assess whether the social and structural determinants of household member support are associated with inequalities in ART adherence outcomes. By considering these aspects, this review provides recommendations for public health interventions aiming to improve ART adherence. 

## 2. Methodology

### 2.1. Search Process

The review methodology followed the recommendations of the PRISMA (Preferred Reporting Items for Systematic Reviews and Meta-Analysis) guidelines for systematic reviews [49]. To identify studies addressing the social and structural determinants of household member support for ART adherence, seven databases (PubMed, Cochrane Library, Embase, the Cumulative Index of Nursing and Allied Health (CINAHL), Medline, Psych-Articles, and Web of Science) were searched on 4 September 2019, for peer-reviewed literature published between 2003 and 2019. Search terms for all databases are detailed in Supplementary 1. Search terms were adjusted according to each database (see Table 1 for an example). One author (LC) conducted the search, which was checked by another two authors (ET, EA). The terms searched thematically covered (1) ART adherence, (2) household and family, and (3) support and care. Social and structural determinants were not explicitly included in search terms given the wide range of terms associated with these factors across different disciplines. 

### 2.2. Inclusion and Exclusion Criteria

Articles were included if they contained data or text assessing how social and structural factors determine or affect household or family support for another family member’s ART adherence. In line with Niehof, this review drew on Rudie’s definition of the household as a “co-residential unit, usually family-based in some way, which takes care of resource management and primary needs of its members”, acknowledging the complexity of the concept and its overlap with that of the family (Rudie in [50], p. 490). This review therefore included articles that contained or focused upon analysis of adherence support from a co-residing household member, who may or may not be a family member. Articles were, furthermore, only included if they focused on population groups that were broadly representative of the general population. Studies were therefore excluded if they referred to high-risk groups, including commercial sex workers, drug and alcohol users, and prisoners. Sexual orientation was included. If a study detailed an intervention, then this was included if it were household/family-focused (even if facility-based), intervening to improve ART adherence, and included analysis of the social determinants of that household’s support. Studies were included if they were set in LMICs, based on the World Bank classification [51]. Adults, children and adolescents were all included in order to examine vulnerabilities and health inequalities by age. Articles were retained if they were in English, French, Dutch, German, or Spanish.

### 2.3. Screening Process 

Following manual and automatic deduplication, six authors (LC, ET, EA, NS, CM, EW) screened the search results for potentially eligible studies by title and abstract according to the inclusion and exclusion criteria. Articles excluded at this stage were set in a high-income country or regarded key groups such as drug users. All retained abstracts were then assessed by a second author; any disagreements were resolved by discussion and, if necessary, by referral to a third author. A list of 168 potential articles were assessed by full text by at least three authors (LC, ET, EA, NS, AD); divergent views were resolved by discussion. The reference lists of relevant articles were searched to find additional studies and were screened by three authors (AD, NS, LC). The final potential articles were then read by three authors (LC, ET, EA), and decisions regarding inclusion were resolved through consensus. Reasons for exclusion included: the study analysed either structural factors or family support for ART adherence, but did not (clearly) link the concepts (111 studies; 82%); interventions did not take family member support into account or did not take structural factors into account (4; 3%); studies did not fulfil the methodology criteria (5; 4%); studies were based in high-income countries (7; 5%); studies did not refer to the general population (7; 5%); full text not found (1; 1%). 

### 2.4. Data Extraction, Assessment of Study Quality and Data Synthesis

A data extraction spreadsheet was developed, detailing intervention and study characteristics, as well as which social determinants for household support are discussed, the reported effect on household support, the type of support given to PLWH, and the reported effect on ART adherence. Five authors extracted data (ET, AD, EA, LTD, TKF, LC), which were checked by a second author (LC, ET). The qualitative studies were assessed by three authors independently (all assessed by LC, plus ET, EA, CM, LTD, AD, and NS) using the guidelines from Spencer et al. (2003) [52]. Three authors independently assessed the quantitative (HT, LC and TKF) and mixed methods studies (EW and LC with input from HT, AD, ET and LTD). The quantitative studies were evaluated using the National Institutes of Health (NIH) Quality Assessment Tool for Observational Cohort and Cross-Sectional Studies systematic review [53], and the mixed methods by using the Mixed Methods Appraisal Tool (MMAT) [54]. Studies were evaluated against the indicators suggested by the guidelines and tools and subsequently assessed as being of good, fair or poor quality, according to the assessed methodological rigor and risk of bias [52,53,54]; for example, the NIH quality assessment tool advises that a good quality rating be given where risk of bias is low, and a poor quality rating where risk of bias is high [53]. Scoring was not undertaken, nor was it recommended by the quantitative tools. For the qualitative studies, as Spencer et al. (2003) suggested, quality was assessed by examining “the questions and indicators most relevant to the evaluation concerned” (in [52], p. 20). Consensus was reached and disagreements between authors were resolved by thorough discussion. It is, of course, important to acknowledge the complexity of bringing together different types of studies into one single form of categorisation. All 33 studies were included in the review, irrespective of assessed quality. 

A mixed methods synthesis was undertaken, in line with Thomas et al. (2004) [55] in order to benefit from findings from mixed methods, quantitative and qualitative studies. The quantitative studies were initially synthesised using a narrative approach [56]; given the heterogeneity between studies in terms of measures used for ART adherence, social and structural determinants, no formal meta-analysis was conducted. Qualitative studies were synthesised using an iterative process of meta-synthesis, also termed meta-ethnography [57], to identify themes that recurred throughout the data: key themes and quotes were collected (first-order findings) and were categorised into six descriptive (second-order) categories of social and structural determinants associated with household member support: stigma, gender, socioeconomic status, education and health literacy, health system factors, and religious and cultural beliefs. Findings from quantitative studies were integrated into the second-order analysis. Second-order constructs were then generated into a line of argument (third-order analysis), regarding the relationship between structural determinants of support and ART adherence and how these social and structural factors intersect. 

## 3. Results

**Figure 3:**
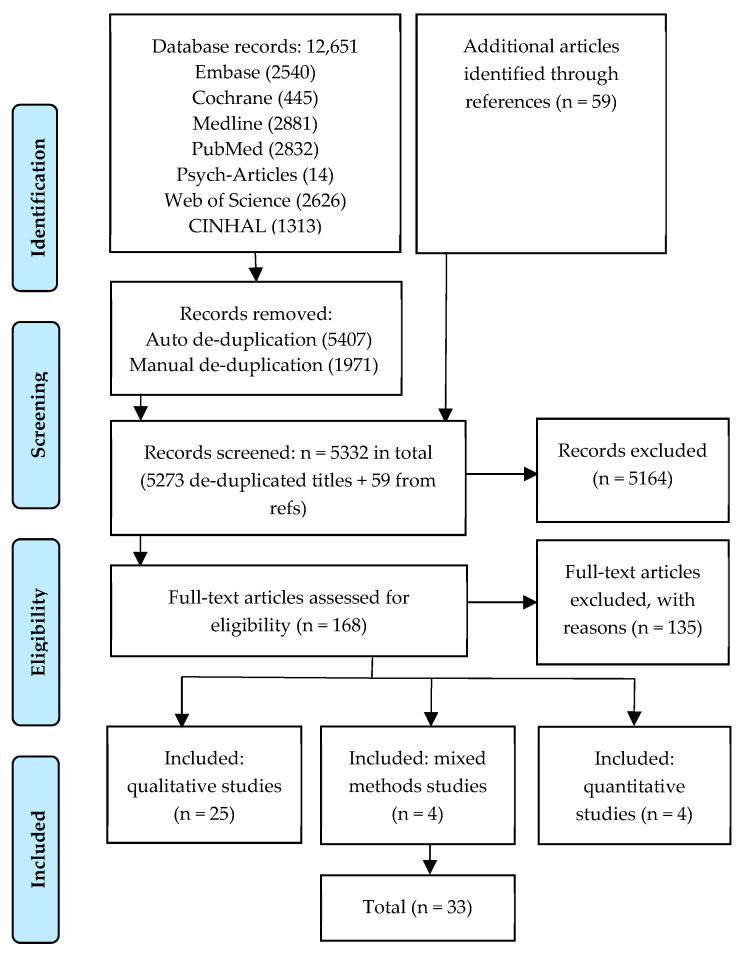
PRISMA flowchart.

The search yielded 12,651 initial records: 7378 were identified as duplicates and subsequently deleted (see Figure 3). The remaining 5273 titles and abstracts were screened to eliminate studies that did not meet eligibility criteria. Bibliographies of articles were searched, and 59 additional texts were retrieved; 5164 articles were eliminated and 168 full-text articles were assessed for inclusion. The review retained 33 peer-reviewed articles published between 2006–2019. 

### 3.1. Study Characteristics

The review comprises 25 qualitative studies [46,58,59,60,61,62,63,64,65,66,67,68,69,70,71,72,73,74,75,76,77,78,79,80,81], four quantitative studies [82,83,84,85] and four mixed methods studies [86,87,88,89], detailed in Tables S1–S3 (see Supplementary 2). Twenty-four studies were conducted in sub-Saharan Africa, including South Africa [58,61,62,65,71,73,74,81,82,84], Uganda [59,67,69,80], Zimbabwe [60,75,77,78], Malawi [46,88], Democratic Republic of Congo [63], Botswana [76], Tanzania [80], Kenya [79], Nigeria [80], and Togo [85]. Eight were conducted in Asia, including India [64,70], Thailand [68,72,83,89], Cambodia [83], China [86] and Vietnam [87], and one in South America, in Ecuador [66]. Eleven studies focused on adults [46,58,61,64,66,74,75,78,80,83,86]; one of these also included children [75]. Twenty-three studies focused on infants, children and adolescents ranging in age from newborn to eighteen years [59,60,62,63,65,67,68,69,70,71,72,73,75,76,77,79,81,82,84,85,87,88,89]. 

The four quantitative studies, comprising three cross-sectional studies and one cohort study, used a range of statistical methods. Three calculated odds ratios using logistic regression models to assess the association between an exposure, such as education level, and adherence as well as virologic suppression [82,84,85]. One study additionally calculated Pearson’s r correlations and performed an explorative principal component analysis (PCA) [84]. Two studies conducted chi-square tests on measures such as knowledge scores to assess the difference in means between groups [83,84]; for one quantitative study, this was the sole statistical method used [83]. Qualitative studies equally drew on a range of methods, e.g., in-depth and semi-structured interviews [46,58,59,60,61,62,63,64,65,66,68,69,70,71,72,73,74,75,76,77,78,79,80,81]; focus group discussions (FGDs) [64,67,68,74,75,77,78,79]; and ethnographic observation [75,80]. Eight studies used two or more qualitative methods, e.g., interviews and FGDs, or interviews, FGDs and ethnographic observation [64,68,74,75,77,78,79,80]. However, underlying theory and/or the type of qualitative analysis was not always described. Mixed methods studies combined cross-sectional surveys with qualitative methods such as semi-structured interviews [86], focus groups [87,88] or in-depth interviews [89]. 

### 3.2. Quality Assessment 

Study quality was assessed ranging from poor to good. Most studies fell in the ‘fair’ category: this broad category covered a wide range of studies, from those that were rich in analysis yet had not noted certain details, to studies that contained many good elements but whose analysis was descriptive rather than analytical. For qualitative studies, important missing sections included acknowledgement of limitations, or a lack of detail on methodology or theoretical framework. Quantitative studies and mixed methods studies suffered from small sample sizes, selection bias, or the use of methods for assessing adherence that were prone to recall bias. Table 2 indicates the assessed quality of evidence available for each social and structural determinant. The best available evidence was on socioeconomic status (SES) and gender. 

### 3.3. Definitions, Concepts and Measurement 

The results reveal that studies drew upon the concept of the family rather than the household, and there was heterogeneity in the measurement of ART adherence, social and structural factors, and the definition of support. 

#### 3.3.1. Definitions, Concepts and Measurement 

The majority of the reviewed studies drew on the concept of the extended family rather than that of the household, distinguishing between those who were family or ‘other’. Family included spouses, step-parents, and biological relatives, such as parents and grandparents. ‘Others’ who supported the ART adherence of PLWH included friends, community members, guardians, or caregivers at orphanages in loco parentis [59,63,67,70,71,72,74,80,85,86,87]. Family co-habitation in households was often implicitly assumed, especially in studies regarding caregivers of children. Only three studies explicitly specified whether family members belonged to the same household as the PLWH, and noted that ART adherence support was facilitated when families lived together [83,85,86]. 

This review, furthermore, makes use of Heaney and Israel’s (2008) categorisation of types of support as instrumental, emotional and informational; this definition is also used by two of the reviewed studies [29,61,69]. All thirty-three articles mentioned family members of all ages (bar infants) providing instrumental support to PLWH. Instrumental support included fetching medication for patients, accompanying patients to clinic visits, financial support, helping with household chores, discussing regimens with doctors, nursing care when ill or for young children, and preparing food for medication adherence. Sixteen articles detailed emotional support being given to all age groups, [46,58,60,61,62,64,66,68,69,70,71,74,75,78,80,86]. Emotional support comprises care and love, including supporting grieving family members who have lost a parent, child or spouse. Five studies discussed informational support [61,67,74,80,86], which includes giving guidance and advice. Fifteen studies examined the kind of support received by caregivers and household members (see Section 3.3.3).

The measurement of adherence in all three types of study varied widely. Two quantitative studies included measurements of viral suppression [82,84]. One included a caregiver report of missed doses in children [85], and one quantitative and one mixed-methods study focused on analysing support and so did not include a quantitative measurement of adherence. These studies instead focused on respondents’ perceptions of improved or worsened adherence [83,87]. One mixed methods study used self-report measures of adherence [86]. Two mixed methods studies used caregiver reports of children’s or adolescent’s adherence [88,89], with one combining adolescent report, caregiver report and viral load measurement [89]. Only seven qualitative studies included discussion about levels of adherence and/or missed doses [58,59,61,63,69,70,73]. Two of these qualitative studies included a measure of viral load or sampled participants based on measurements of viral load [69,73]. The remaining qualitative studies discussed adherence in general terms. 

Measurement of social and structural determinants between studies was inconsistent. For example, variables affecting socioeconomic status, such as income, education, and occupation, were most often regarded as demographic variables rather than being examined as a composite measure of socioeconomic status. Two studies included access to sanitation and assets [82,84], one measured the difficulty of the financial situation [89] and two included monthly income [84,86]. Education was measured either in years [83,84] or in level of achievement, such as completion of secondary school or primary school [82,85,86,87,89]. 

#### 3.3.2. Social and Structural Determinants Affecting Household and Family Member Support for ART Adherence

The results indicate that household support for ART adherence was affected by the following structural and social factors: gender, education, religious and cultural beliefs, socioeconomic status, stigma, and health system functioning. Furthermore, household members drew upon networks of support to enable them to overcome social and structural barriers to support PLWH’s adherence to ART. Results are summarised in a figure in each section, outlining how structural factors affect household support, and in turn adherence. Dashed lines between boxes indicate where studies have measured the association between structural factors and adherence directly. 

##### Gender 

**Figure 4:**
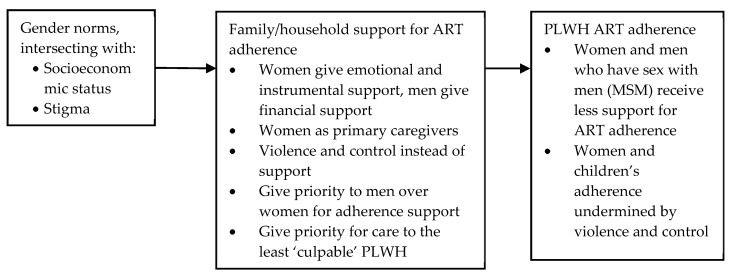
Studies examined the indirect impact of gender norms on ART adherence via household support.

Ten studies explored the impact of gender norms on household member support for ART adherence (see Figure 4), based in South Africa [58,61,62,81], Zimbabwe [75,78], Malawi [46,88], Ecuador [66] and India [64]. Gender norms affected household members in terms of who is expected to give support and the type of support given; gender norms affected PLWH, of all ages, in terms of whether they receive support at all and the quality of care they receive. 

In five studies, gender norms determined who gave support and the type of support given to PLWH: two studies in Malawi (on couples with at least one partner on ART; children and their caregivers in the Zomba district), two in South Africa (on women in KwaZulu-Natal caring for a child with HIV; men in mining communities and their long-term live-in partners), and one in Ecuador (on PLWH, their relatives, and health care providers) [46,58,62,66,88]. In these studies, gender norms positioned women as primary caregivers, resulting in inequalities between male and female household members in terms of the allocation of labour and time for care—exacerbated by the introduction of the need for HIV care [46,58,62,66,88]. Men primarily gave instrumental (financial) support, while women gave (and expected to receive) both instrumental and emotional support for family members’ ART adherence [46,58,66]. 

Six studies in Zimbabwe, South Africa and Malawi explored how hegemonic notions of gender and masculinity, combined with HIV stigma, prompted male household members to actively undermine women and children’s ART adherence [46,61,62,75,78,81]. In two studies located in Manicaland, eastern Zimbabwe, male ARV users stated that identifying as having HIV threatened their masculine identity by forcing them to appear to be feminine [75,78]; they associated illness with needing support, and needing support and asking for help were considered to be feminine attributes [78]. Women and healthcare workers reported that, instead of offering support, male partners (who refused to get tested themselves or disclose their HIV status) would exert control and be violent. Men prevented their partners from going to the hospital to get drugs or stole their ARVs to use themselves in secret [75,78], threatened divorce if they used ARVs, insisted on not using condoms and thereby threatened them with re-infection [78], or prevented them from attending clinics or support groups [75]. Similarly, in two studies on couples in Malawi (with at least one partner on ART) and KwaZulu-Natal, South Africa (the majority of couples seroconcordant and 63% with both on ART), threats and acts of violence and control such as telling female partners not to take their ART affected women’s physical and mental health, and meant women refrained from asking partners for support [46,61]. As women were financially dependent on partners, they were unable to access treatment without financial support [75]. Children risked being impacted by the mother’s financial dependency: a study among caregivers of children in Gauteng province, South Africa, reported that women could not take children to the clinic for fear of upsetting the father, as the mother’s (and thereby father’s) positive HIV status was often inferred from the child’s [81]: mothers feared being blamed for infecting the child and subsequently being abandoned by their partner, with the consequent loss of financial support [62,81]. However, two studies on couples in Malawi and South Africa indicated that where relationships were more equal, support for ART adherence, including mutual support where both partners were living with HIV, could strengthen couple dynamics [46,58]; furthermore, Conroy et al. (2018), in a study in Malawi, suggested that women may gain a certain amount of power from their partner’s need for support as it enabled women to negotiate fidelity in return for providing love and care—although wives were nevertheless obliged to take care of husbands if they wanted to remain married [46].

Gender norms varied across cultural contexts in determining who received adherence support by giving priority to certain PLWH based on their gender, sexual orientation or financial contribution to the household. In a study in India, men were prioritised for family support for adherence even when household resources were limited: keeping them healthy meant they could earn a living for the family [64]. Women, however, reported receiving little to no support for adherence from family and household members because they were not considered to economically contribute to the household [64]. In contrast, a study in Ecuador examined how the amount of support for ART adherence given by family members was greatest for those who had acquired the virus in the least ‘culpable’ manner [66], and lowest for those who were most ‘culpable’. Intersecting gender norms and HIV stigma led to a perceived scale of ‘best’ to ‘worst’ modes of HIV transmission: the greatest amount of support, and the best quality of support, was given to those who had acquired the virus via the ‘least culpable’ method of transmission such as blood transfusion or at birth. The least amount of support was given to those who had acquired HIV via the ‘most culpable’ route of transmission, which was understood in this context to be men who have sex with men (MSM). In-between were married women acquiring HIV from an unfaithful husband, heterosexual men who have occasional casual sexual relations, and single women without a stable relationship. 

##### Education: Literacy, Language Skills and Health Knowledge

**Figure 5:**
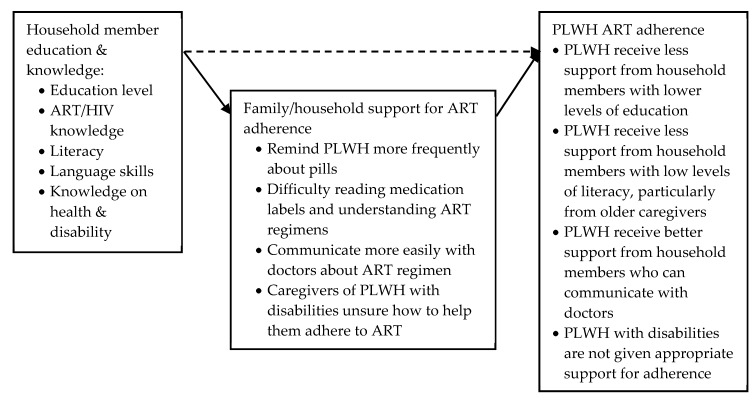
Studies either measured a direct relationship between household member education level and the ART adherence of people living with HIV (PLWH) or examined how this relationship operates via household support.

Thirteen studies examined the multifaceted relationship between education, household support and ART adherence in studies from South Africa, Zimbabwe, Botswana, Cambodia, Togo, China, Vietnam and Thailand (see Figure 5) [65,74,75,76,77,81,82,83,84,85,86,87,89]. Quantitative results focused on caregiver education level and children living with HIV; four studies (three quantitative and one mixed methods) measured a direct relationship between caregivers’ education level and ART adherence of children or adolescents (dashed arrow in Figure 5) [82,84,85,89]. While one quantitative study based in South Africa found some evidence of an association between caregivers’ secondary education level and ART adherence (OR = 4.49, (1.10–18.24)) [82], the other three studies based in South Africa, Togo and Thailand found no association [84,85,89]. Further qualitative and quantitative results suggested, however, that the pathways between household member education, support and ART adherence of PLWH (adults and children) revolved around three complex interlinked issues: household member health knowledge, literacy, and language skills.

Six studies described the relationship between education, HIV/ART knowledge, and support for adherence [62,81,82,83,85,87]. Two studies that measured the direct relationship between caregiver knowledge of HIV and ART and PLWH’s ART adherence (dashed arrow in Figure 5) found no association between the two [82,85]: in a study in Togo, ART adherence was not associated with caregiver knowledge of HIV [85], and in a study in Cape Town, adherence was not associated with caregivers’ knowledge of the ART regimen—but was associated with caregivers’ education level [82]. Two other studies examined the indirect pathways between caregiver education, knowledge, support and adherence (black arrows in Figure 5). In a quantitative study in Thailand and Cambodia, caregiver education level was significantly associated with correct knowledge of ART in Cambodia (*p* < 0.05), but not in Thailand [83]. Caregivers with better knowledge of ART, indicated by higher ART knowledge scores, reported reminding their adult children and adult grandchildren to take their ARVs more frequently than caregivers with lower ART knowledge scores [83]. Similarly, caregivers in a mixed-methods study in Vietnam explained that low knowledge of ART medication and regimen negatively impacted how they could support their household member to adhere to ART [87]. One qualitative study in South Africa indicated that caregivers’ lack of knowledge on HIV/AIDS prevented children from even being tested or accessing care as caregivers saw HIV as being primarily a sexually transmitted infection, and therefore not relevant to children [81]. Similarly, a study in South Africa among caregivers with low levels of education (a few years’ primary education) stated that mothers would take their child off ART if the child was perceived to be healthy and doing well—although it was not stated if these mothers were themselves on ART [62].

Caregiver ART and HIV knowledge was linked to a caregiver’s own HIV status in two studies [71,85]. Peterson et al. (2010) noted that two adolescents were very well informed about HIV as a result of their caregivers also living with HIV [71]. In a study in Togo, Polisset (2019) stated that children were more likely to be adherent if their caregiver did not perceive difficulty in ARV administration [85]. Interestingly, in chi-square tests, the percentage of caregivers who demonstrated little knowledge of HIV/AIDS and the percentage of caregivers who perceived difficulties in administrating ARVs “did not differ in whether they had declared they were HIV-positive or not” (in [85], p. 26). 

Four studies observed a relationship between education, caregiver literacy and PLWH adherence, in South Africa, Zimbabwe, Botswana and China: limited education results in low levels of literacy, which meant that caregivers had difficulty reading medication labels, appointment cards and understanding ART regimens [65,75,76,77,86]. Three studies linked illiteracy to age, with older caregivers having lower levels of education, lower levels of literacy, and consequently particular difficulties in supporting ART adherence for children and grandchildren [75,76,77]. One study in South Africa furthermore linked low levels of education and literacy with a lack of appropriate health knowledge around disability; this study noted that caregivers’ lack of knowledge regarding how to deal with children with additional learning needs was a considerable obstacle to effective adherence support for the child [65].

One study stated that improved English language skills meant an increased ability to discuss ART regimens with doctors, who may not be fluent in the caregiver’s local language: Muller et al. (2011) found that caregivers’ confidence in speaking English with the doctor was significantly correlated with viral suppression in their household member in South Africa [84]. Muller et al. furthermore developed a factor model in which the caregiver's language skills, child's health status, disclosure of caregiver's status and perceived stigmatisation explained 95% of the variation in viral suppression [84]. 

##### Religious and Cultural Health Beliefs 

**Figure 6:**
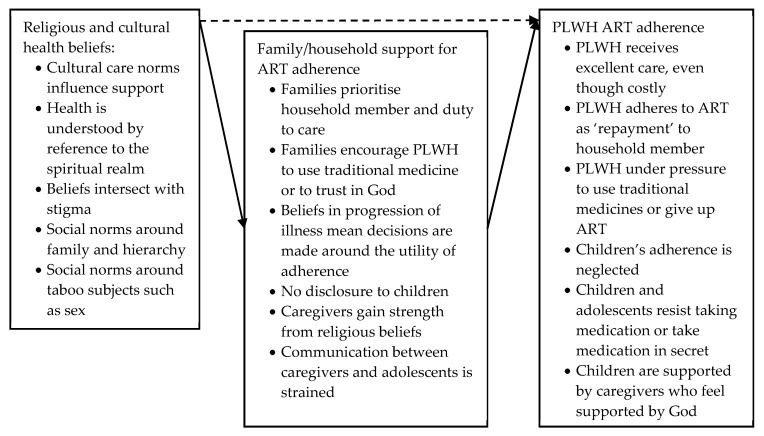
Studies either measured the impact of religious, cultural and health beliefs on the ART adherence of PLWH, or examined the ways in which these beliefs impact ART adherence via household support.

Eleven studies located in South Africa, DRC, Botswana, Kenya, Thailand, China and Ecuador acknowledged that household support for ART adherence is strongly influenced by cultural context, religious beliefs, and social norms (see Figure 6) [62,63,66,68,76,79,80,81,82,86,89]. Importantly, the type of care available for PLWH is based on cultural care norms that vary between communities, countries and continents. For example, Fredriksen-Goldsen et al. (2011) described how care norms in China, a collectivistic society that emphasises the needs of the family above those of the individual, led to families driving themselves into debt in order to care for their household member living with HIV [86]. In other contexts, care may form part of systems of exchange: Ware et al. (2009) describe how caregivers in South Africa expected adherence as ‘repayment’ for their support, with PLWH adhering to ART in order to promote goodwill and ensure help would be available in the future [80]. 

Five studies examined how beliefs that understand health by reference to the spiritual realm affected PLWH’s ART adherence either positively or negatively [62,63,66,76,79]. Fetzer et al. (2011) stated that religious faith gave caregivers the strength to carry on caring for their children and supporting their ART adherence [63], whereas other studies mentioned that household members encouraged PLWH to turn away from conventional medicine and to look to either religious faith or alternative health systems, such as traditional medicine [62,66,76,79]: for example, a study in Ecuador stated that religious faith led to household members encouraging adult PLWH to give up ART [66]. Studies in Kenya, Botswana, and South Africa gave examples of how cultural and religious beliefs about health intersected with HIV stigma: the studies referenced cultural health beliefs that attributed HIV to witchcraft, noting that the resulting stigma led to non-disclosure of a child’s HIV status, and the concealment of medication or the preferential use of traditional medicines [62,76,79]. One study based in South Africa stated that some caregivers believed that knowing a child’s HIV status would cause the illness to progress faster, prompting neglect of the child’s adherence [81]. A quantitative study in Cape Town, South Africa, however, did not find any direct quantitative association between caregiver health beliefs and child ART adherence (dashed arrow in Figure 6) [82]. 

Two studies in Thailand indicated that Thai cultural and social norms regarding communication affected caregiver support for adolescent ART adherence [68,89]. Nestadt et al. (2018) identified caregivers’ key concerns around inter-generational communication as an impediment to effective support: social norms around family hierarchy meant that children and adolescents were expected to follow caregivers’ orders regarding ART adherence without question, straining communication and relations between generations [68]. Xu et al. (2017) noted that communication worsened when caregivers were not the biological parents of the child on ART and that this impacted adherence (aOR for suboptimal adherence if the caregiver is “other family members”: 2.100 (CI 1.277 ± 3.456), *p* = 0.003) [89]. Both studies furthermore noted that difficulties around communication extended to other HIV-related topics, such as sex education or stigma [68,89]. Similarly, a study in Botswana noted that social norms dictating secrecy around sex impeded the disclosure of a positive HIV status to children: the lack of disclosure made supporting adherence more difficult [76].

##### Socioeconomic Status: Poverty

**Figure 7:**
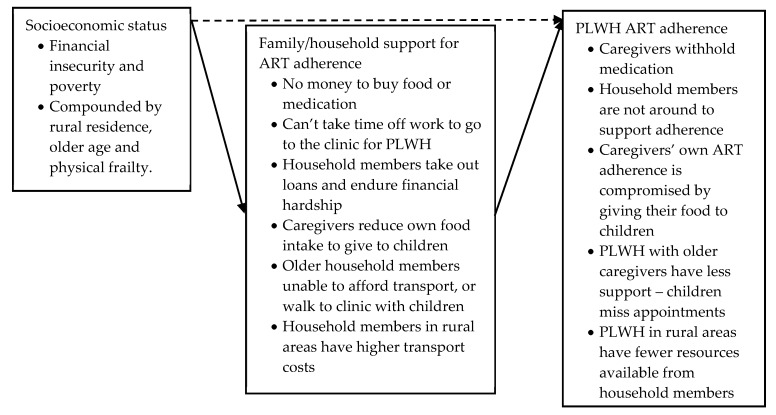
Studies either measured the direct impact of socioeconomic status on ART adherence or examined how socioeconomic status impacts ART adherence via household support.

Low socioeconomic status was the most frequently discussed factor that affected household member support for PLWH’s ART adherence, mentioned in twenty-six studies located in Uganda, Zimbabwe, South Africa, DRC, Botswana, Kenya, Nigeria, Malawi, Tanzania, Togo, India, Thailand, China and Vietnam [59,60,62,63,64,65,67,68,69,70,71,72,73,75,76,77,79,80,81,82,84,85,86,87,88,89]. Quantitative studies mostly measured direct relationships between household socioeconomic status and ART adherence of PLWH (dashed arrow in Figure 7), whereas qualitative studies explored the indirect relationship between poverty and adherence by examining how this impacted household support for ART adherence (black arrows in Figure 7). While one quantitative study in South Africa with caregivers of children found evidence of a significant statistical association between household socioeconomic status and ART adherence (household access to water and electricity as a predictor of annual MR adherence ≥ 90%, chi-square test *p* = 0.024, OR = 2.65, (0.93–7.55)) [82], other studies located in Thailand and China among adults and adolescents did not [86,89]. The study in China, furthermore, found no evidence of a significant association between PLWH’s adherence and household socioeconomic status, or adherence and caregiver support [86]. One study in South Africa on caregivers and young children found evidence of a significant association between owning a mobile phone and adherence measured by microchip recording of opening the medication (chi-square test *p* = 0.01), but not viral suppression (chi-square test *p* = 0.12), while other variables such as access to water or electricity were not associated with either adherence or viral suppression [84].

Low socioeconomic status meant limited financial resources available to support a household member living with HIV. Studies undertaken during periods when ART was not widely available for free documented household members’ difficulty affording out-of-pocket healthcare costs when supporting PLWH: for example, Weigel et al. (2009) note that their research took place when ART was not free in Malawi, and low socioeconomic status meant that caregivers had to reduce the dose they gave their child to make the drugs last longer [88]. In a study in China, Fredriksen-Goldsen et al. (2011) stated that under certain policies ART was free, but many patients were ineligible: families would therefore drive themselves into debt to care for their (adult) household member living with HIV [86]. Punpanich et al. (2008), in a study in Thailand, also noted that caregivers were concerned about affording ART when adolescents turned 18 and transitioned into ‘adult’ care [72]. 

The global scale-up of free ART has not, however, completely alleviated the impact of socioeconomic status on household member support for ART adherence. Scott et al. (2014), in a study among adults and children in Zimbabwe, stated that it was still necessary to pay for medication for opportunistic infections, while Skovdal et al. (2011) stated that the cost of clinic visits was a concern for grandparents caring for children [75,77]. In later studies undertaken in Thailand (on adolescents) and Malawi (adult couples) when ART was widely available for free, concerns around socioeconomic status centered on caregivers having to earn an income and purchasing food for partners [46,68]: in total, twelve studies in Uganda, Zimbabwe, South Africa, DRC, Kenya, Malawi and Vietnam dating from 2006–2017 reported that household members had limited resources to pay for food for PLWH to eat when taking medication [59,60,62,63,69,71,73,77,79,80,87,88]. One qualitative study on children in Zimbabwe, however, stated that food insecurity was nevertheless not considered to be a barrier to adherence [60]. Weigel et al. (2009) stated that when ART was made free in Malawi, demand rapidly increased which lengthened waiting times at hospitals which was a problem for caregivers: household members in Zimbabwe, Kenya, South Africa and Vietnam also stated that they had to work in order to ease financial difficulty but long waiting times at the clinic meant the opportunity cost of accompanying PLWH to appointments was high [60,62,87], and required flexible arrangements with employers [79]. Equally, in studies in Thailand and Uganda, having to work and not being present at home meant that household members could not adequately care for children or adolescents by giving reminders or overseeing medication adherence [67,68]. 

The reported relationship between socioeconomic status and education was complex. Davies et al. (2008) in a study in South Africa stated that having secondary education was not significantly associated with any of the variables associated with socioeconomic status, such as electricity or water [82]. Low levels of education compounded financial hardship in a study in Vietnam: caregivers with education levels lower than high school reported greater financial burden compared to those with high school level or over (OR = 2.08, (1.50–4.20), *p* = 0.02) [87]; the study did not clarify, however, how it coded and analysed participant responses to their ‘financial burden’ questions into “greater financial burden”. 

Socioeconomic status intersected with other factors such as residential location and age to create greater difficulties for household members supporting ART adherence. Older caregivers in a study in Zimbabwe emphasised the particular economic difficulties they faced, as their lower levels of education and greater physical frailty prevented them from working or attending the clinic– which forced often ill children out to work instead [77]. Rural residence was cited to be a particular issue for these older caregivers, who could not afford transport and could not physically carry small children to the clinic or even walk the long distances themselves in order to collect ART medication [77]. Caregivers of children in three other studies in Kenya, Vietnam and Malawi also stated that household members living in rural areas faced higher transport costs due to the longer distances travelled to the clinic, which presented a considerable barrier in attending appointments to collect medication [79,87,88]. Household members based in urban locations close to the clinic, however, were not affected by the cost of transport which therefore facilitated clinic visits [79,87,88], and in a study in China conducted with adult family members, urban residence was associated with ART adherence [86]. Similarly, Tran et al. (2017) stated that rural caregivers in Vietnam reported significantly higher ‘financial burden’ than urban caregivers (OR = 2.26, 95% (1.24–4.11), *p* = 0.007) [87].

Household members, therefore, faced a series of stark choices and competing priorities for household expenditure, decisions which could potentially impact their household member’s ability to adhere to ART. Parents in Zimbabwe and Uganda would withhold drugs from children or reduce doses to avoid medication side-effects when facing food shortages [60,67]. Caregivers of children in Uganda would go without food themselves so that children would eat, which also endangered the ART adherence of caregivers who were themselves living with HIV [69]. Three studies in Uganda and China reported that household members took out loans or credit in shops and banks to financially support PLWH in the household, both adults and children, which caused financial stress and economic hardship [69,80,86]. 

##### Stigma: Secrecy, Disclosure and Access to Support

**Figure 8:**
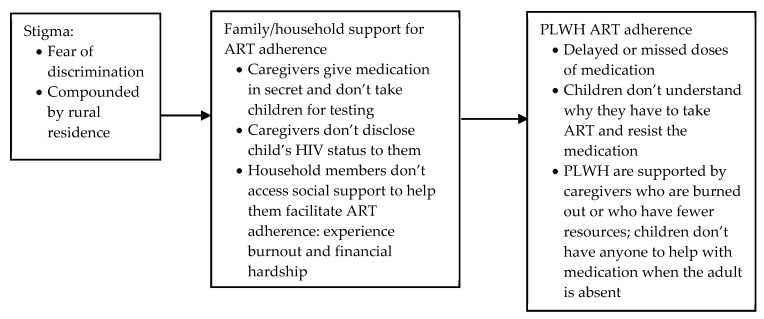
Impact of stigma on household support and, consequently, ART adherence.

Twenty-three studies explored how fear of stigma affected adherence support given and received, by household members, in India, Thailand, China, Vietnam, Ecuador and every country in sub-Saharan Africa included in the review (see Figure 8) [59,60,62,63,65,66,67,68,69,70,71,72,73,75,76,79,80,81,84,85,86,87,88]. Stigma affected household member support in three ways which impacted ART adherence for PLWH: testing and medication; disclosure to children; and accessing social support. 

First, stigma affected medication, testing and access to health care. Stigma around HIV meant that household members feared discrimination, social ostracism and abandonment if their household member’s HIV status became known (and, thereby, their own possibly revealed, if the household member was also living with HIV). If the PLWH was a child, the caregiver would, therefore delay or miss giving ART doses if visitors or other family members were present [60,63,65,73,76,79,84]. Two studies in South Africa furthermore reported that if the child’s caregivers were themselves living with HIV, caregivers consequently avoided seeking health care for their child or having their child tested as the caregiver feared being blamed and abandoned [62,81]. This is particularly important given children’s vulnerability in their absolute dependence on their caregiver for ART adherence. 

Second, studies in Zimbabwe, South Africa, DRC, Kenya, India and Thailand indicated that HIV stigma and the need for secrecy meant that caregivers may withhold from disclosing the child’s status to them [60,63,65,70,72,73,79]. Non-disclosure meant that caregivers encountered difficulties in persuading children and adolescents to take a medication that they did not understand, and had to resort to beatings, manipulation and persuasive techniques to ensure adherence [60,63,65,70,72,73,79]. However, a strong child–caregiver relationship was noted to support adherence even when disclosure had not been made [59,63,69,70]. Disclosure was complicated by the fact that many caregivers were themselves living with HIV, or were looking after a child following the death of one or both parents due to HIV. In a study in Botswana, Shaibu (2016) found that only four out of the twelve grandmothers of orphaned children had told their grandchildren that their mothers had died of HIV; the rest had not disclosed the cause of death and felt uneasy at the thought of disclosing the deceased mother’s positive HIV status [76]. However, in a study in South Africa, Petersen et al. (2010) reported that caregivers revealed that disclosure to a child about their status was easier if the caregiver was also living with HIV [71]. Whilst Polisset et al. (2009), in a study in Togo, found no association between disclosure of child’s HIV positive status and adherence, they also found that children whose caregiver had declared that they were living with HIV were more likely to be adherent (correlate of non-adherence to ART for caregiver’s reported HIV infection OR = 0.4, (0.1–1.0) *p* < 0.05) [85]. A study in South Africa by Muller (2010) developed a factor model with a child's health status, caregiver’s language skills, disclosure of caregiver’s status and perceived stigmatisation, which explained 95% of the variation in viral suppression of the child [84].

Third, secrecy around the PLWH’s status (whether the PLWH was a child or adult) meant that household members were prevented from asking their social networks for help with supporting PLWH’s ART adherence [60,62,63,67,69,71,72,76,79,84,87,88]. Family members and caregivers could not give a ‘good reason’ for borrowing money for transport/food, they could not participate in support groups or home-based care groups [60,63] and did not have enough emotional support from family—which led to feeling stressed and burnt out, affecting their ability to support the PLWH in the household [62,67,71]. Additionally, in the case of children and adolescents, if the caregiver was at work or otherwise engaged away from home, they could not ask other adults to support the child or adolescent to take their medication at the appropriate time [71,72,79,87,88]. Tran et al. (2017), in a study in Vietnam, stated that caregivers living in rural regions reported experiencing higher levels of stigma (OR = 3.53, 95% CI 1.36–9.06, *p* = 0.006), meaning less recourse to financial support through support networks [87]. These difficulties were compounded for those caregivers living with HIV themselves; for example, a study in Zimbabwe stated that due to stigma, caregivers living with HIV were less likely to disclose either their own or the child’s HIV status, and would thereby access less social support for either of them [60].

##### Health System Factors: Trust and Functioning 

**Figure 9:**
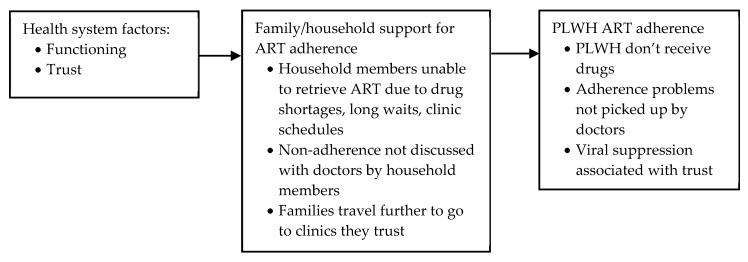
Impact of health system factors on household support and, consequently, ART adherence.

Eleven articles discussed how (trust in) the functioning of health systems affected household member support for ART adherence, in studies based in South Africa, Zimbabwe, Malawi, Togo, Uganda, Tanzania, Nigeria, Vietnam and India (see Figure 9) [60,67,70,73,79,80,81,84,85,87,88]. Four studies from sub-Saharan Africa mentioned that drug shortages prevented household members from picking up ART medication for the PLWH in the household [60,73,85,88]. Long waiting times and queues, inflexible clinic schedules with visits during school/working hours and ART pick-ups that required the presence of the PLWH were cited as barriers to obtaining timely doses of ART for household members in five studies from sub-Saharan Africa and one study from Vietnam [60,67,73,80,81,87]. Furthermore, four studies from sub-Saharan Africa indicated that unexpected early closure of pharmacies, constant changes in the labelling of the medication, and castigatory modes of communication from health providers destroyed trust in the health service and in medicine itself [60,73,79,81]. The non-adherence of household members was therefore sometimes not discussed with health service providers, missing vital opportunities to obtain support [79]. In India, a qualitative study reported that trust in the health system was undermined by a lack of faith in government, meaning that people did not trust the quality of the free ART available at local clinics; this, combined with a fear of stigma if recognised at local clinics, prompted families to travel further to purchase ARVs from private clinics (putting a further strain on family finances) [70]. Muller et al. (2011) undertook an exploratory PCA to examine correlates of adherence in South Africa. The first factor, ‘trust and coping’ correlated significantly with caregiver quality of life (r = 0.42; *p* <0.01) and viral suppression (r = 0.34; *p* < 0.01), indicating a link between viral suppression and trust in the doctor [84]. 

#### 3.3.3. Caring for PLWH and Caring for the Carers

In order to support PLWH to adhere to ART, their household members and caregivers also require help and care: for example, Muller et al. (2011) found that whether caregivers had “enough time to look after [their] own health” was significantly associated with virologic suppression in children [84]. Fifteen studies in Uganda, Zimbabwe, DRC, South Africa, Botswana, Kenya, Nigeria, India and Cambodia indicated that social support for household members of PLWH meant they could overcome the challenges presented by social and structural factors such as low socioeconomic status and low levels of education [59,60,63,67,69,70,71,74,75,76,77,79,80,83,84]. For example, in Uganda, South Africa and Tanzania, when household members of PLWH drew on strong networks of relationships, social support enabled them to access food, money, transport and childcare [69,71,80]. A study in Uganda interviewed caregivers of both virally suppressed and non-virally suppressed children: while caregivers in both groups received social support, the reported level of instrumental support was greater among the caregivers of virally suppressed children, featuring more conspicuously in discussions [69]. Similarly, in a study in South Africa, Petersen et al. (2010) stated that caregivers who were experiencing difficulty in coping were either living on their own or were living in an unsupportive family arrangement [71]. For household members living with HIV themselves, the importance of social support is vital; for example, in a study in Kenya, Vreeman et al. (2009) stated that caregivers’ poor health due to HIV could affect child adherence, indicating the importance of accessing support for both members of the household, especially during periods of illness [79]. Vreeman noted, however, that care for carers may be culturally defined, stating that different ethnic groups held varying beliefs about the level of care to be given to households where PLWH resided, which overlapped with HIV stigma, and that the key to being given care was having the support of the community elders [79].

Household members of PLWH may obtain support from several different sources. Support for household members may come from the PLWH themselves. When children had higher literacy levels than parents or grandparents, they read the medication labels to them, interpreted information for them and reminded caregivers to take them to appointments [75,76,77]. Children also worked to contribute money to the household [75]. Support may also be external to the household: Knodel et al. (2011) suggested that information on ART from external organisations can overcome educational difficulties [83]. An intervention designed to improve ART adherence, detailed by Remien et al. (2013), used multimedia tools in order to overcome the challenges associated with low education and literacy levels of household members and patients [74]: household members stated they appreciated the visual tools and that they could better support their household member as a result of the knowledge gained through the intervention [74]. Petersen et al. (2010) stated that caregivers who are grieving the loss of family members to HIV, whilst also caring for orphans, may benefit from therapeutic interventions [71]. 

## 4. Discussion

This systematic review aimed to review the literature examining social and structural determinants of household member support for PLWH’s ART adherence within households and families in LMICs, identifying 33 studies conducted in 15 LMICs. Whilst previous reviews have mentioned that social and structural factors affect household support, this study has identified current evidence on the modalities by which this takes place. This review has also contributed to the literature by examining whether the social and structural determinants of household member support are associated with inequalities in ART adherence outcomes. The findings indicated a set of causal pathways between structural and social factors, household support, and PLWH’s ART adherence, suggesting that variation in the risk of poor ART adherence across different groups may be partly explained by differences in exposure to household support that is affected by these determinants. This warrants further research. Measurement of the impact of social and structural determinants on household support for ART adherence is, however, complex. Quantitative analysis in the reviewed studies frequently examined direct relationships between social determinants, household support and adherence, linking the three either via a series of separate measurements or with qualitative analysis [82,83,84,85,86,87,88,89]. The reviewed quantitative studies did not undertake path analysis to examine causal pathways, although qualitative studies examined the paths by which social and structural determinants exert an indirect influence on adherence through household support [46,58,59,60,61,62,63,64,65,66,67,68,69,70,71,72,73,74,75,76,77,78,79,80,81]. The majority of evidence was, however, rated as fair by the quality assessment. 

The findings of this review indicate that household support for ART adherence was affected by social norms around gender, which varied between cultural contexts. In all the relevant reviewed studies, social norms around gender placed the burden of care for PLWH on the shoulders of female household members, a finding echoed in previous literature [90]. For PLWH, however, whether they received household support for ART adherence was affected by a complex and culturally related interplay of gender norms, social norms around care, socioeconomic status and HIV stigma. This is consistent with findings from ethnographic literature that posits that social norms around family care and support for PLWH are context-specific and shifting, dependent on historically changing conditions and norms around stigma and the rights of PLWH [91]. Importantly, hegemonic notions of gender and masculinity that led to gender-based violence from household members were linked to worse health outcomes for female PLWH, which is consistent with previous studies that found a significant association between gender-based violence and lower ART use as well as lower odds of viral suppression [92]. It is, therefore, vital that future interventions take a gender-transformative approach when designing and assessing ART adherence interventions. 

Previous literature has examined a direct relationship between caregiver education levels and ART adherence in children and adolescents in a systematic review in HICs [41], and two reviews in LMICs [38,43], all of which found an association between higher caregiver education level and better ART adherence in PLWH. This review, however, suggests that links between household member education, household support and ART adherence may follow complex pathways that involve language skills, literacy, disability knowledge and HIV/ART knowledge. Similarly, Arage et al. (2014) found a negative association between caregiver education level and PLWH’s ART adherence, yet a positive association between caregiver ART knowledge and adherence [93]. In addition, education is often considered to be an indicator of socioeconomic status, acting as a gauge of knowledge-based assets [94]. The results of this review were inconsistent in this regard. This is unsurprising given that relatively few of the reviewed studies examined the relationship between education and economic assets, as well as the fact that these were set in different countries and populations. Household- or family-based adherence interventions should take into account the education, literacy and language skills of household members, incorporating suitable materials for those with lower levels of education and literacy. 

Religious and cultural beliefs were found to affect household support in a mixture of ways, from positively (giving strength to caregivers of children) to negatively (family reaffirms religious and cultural faith by turning away from medical advice and encouraging PLWH to give up ART), although what constituted religious and cultural beliefs was difficult to define and measure. These results are consistent with a previous systematic review on religion, spirituality and ART adherence, which found mixed evidence concerning the relationship between these aspects: negative associations between ART adherence and religious beliefs occurred when religious beliefs were threatened by coping with illness, and positive associations with ART adherence were found when religion both gave access to social support networks as well as functioning as a literal extension of the support network (God as a supportive figure) [95]. Household-based interventions should therefore take into account the particular religious and cultural context in which they are situated, working alongside religious leaders as well as drawing on positive associations that the religious and cultural context may offer, and developing materials to counter the ‘threat’ that HIV and ART may pose to religious or cultural belief. 

Poverty was the most frequently cited challenge in terms of supporting household members to adhere to ART, although evidence of significant associations between household socioeconomic status and adherence was difficult to measure. Previous studies have also highlighted this complexity: while a systematic review in LMICs found an association between household socioeconomic status and children’s non-adherence to ART [43], a second on adolescents did not [38]. A review by Swann found that interventions such as cash-transfers and transportation assistance showed positive trends for ART care and adherence, with mixed results for clinical outcomes [96]. This review found that factors such as age, education and rural residence intersected with that of socioeconomic status: those who were older, with little education, living in rural areas experienced greater financial hardship. This resonates with other studies that indicate that caregiver age may have an impact on the adherence of children in their care [97]. Those designing adherence interventions should therefore consider how they may mitigate the particular socioeconomic disadvantages faced by household members, particularly the elderly and/or those in rural areas. 

Stigma consistently negatively affected household support for ART adherence in the reviewed studies, in terms of a lack of disclosure to children or community. A systematic review by Aderomilehin et al. (2016) detailed that disclosure to children and adolescents may have a positive effect on their adherence via improved mental health or willingness to take medication, but may also negatively affect mental health and wellbeing [98]. Lack of disclosure to the wider community because of a fear of stigmatisation means that caregivers are unable to access social support and thereby overcome social and structural barriers such as financial hardship. Both qualitative and quantitative studies in this review noted inequalities in adherence outcomes for PLWH according to the amount of social support the caregiver was able to draw upon. Similarly, in a systematic review by Katz et al. (2013), social support was found to be crucial for PLWH themselves in overcoming structural barriers to adherence [99]. Interventions aimed at improving adherence need to support both PLWH and their caregivers in dealing with stigma experienced at home and in the community, in addition to directly tackling HIV stigma via public awareness campaigns or community outreach. 

The findings of this review indicate opportunities for interventions to improve trust between health care providers, caregivers and PLWH, by improving supply chain management and offering training for health personnel in client–provider communication. Health system factors affected household support for ART adherence through drug shortages, missed opportunities to discuss non-adherence and families enduring the extra financial burden of travelling distances to clinics that they trust. Children were particularly vulnerable in this scenario, given their dependence on caregivers for ART adherence. A systematic review of factors affecting adherence among adolescents in sub-Saharan Africa concurred that weak health systems impeded caregiver support for adherence, yet also indicated that where health systems offered support such as peer education support groups, these could facilitate adherence of PLWH [35]. Household-based ART adherence interventions could support access to health care in households where household members are older, more physically frail caregivers for children. It is, of course, important to note local and regional variations in health system functioning when designing adherence interventions; for example, a study analyzing ART adherence in Asia and Africa described the Asian study sites as better resourced compared to those examined in Africa, and that adherence levels in Africa were lower in under-resourced public facilitates than non-public facilities [100]. 

The findings of this review also indicate important gaps in the literature. Although age-related mobility restrictions were discussed as a factor affecting support for adherence, household support predicated on other forms of disability of the caregiver was not examined. Disability was an important barrier to collecting drugs and adherence in a study in Zambia [101] and was negatively associated with adherence in a study in South Africa [102]—and yet only one study in this review mentioned knowledge around disability as a factor affecting household support. Only one study in this review examined how stigma around sexual orientation affected family support for ART adherence, while previous literature in LMICs and HICs has shown that stigma and a lack of social support is an important factor in non-adherence for gay, bisexual, and other men who have sex with men [103,104,105]. In a study analyzing ART adherence in Asia and Africa, authors hypothesized possible under-reporting of MSM among African male participants due to stigma and consequent limited access to services, further highlighting the pressing need to reach this underrepresented group [100].

The results also emphasise the need for good-quality, methodologically rigorous studies. The majority of the quantitative and mixed methods studies were cross-sectional, which limited inference of causal relationships between structural determinants, household support and ART adherence. Furthermore, the quality of the reviewed studies ranged from poor to good, with the majority in the fair category. The results of this review underline the importance of the definition of the family and/or household, as well as measurement of social and structural factors and adherence. Debate surrounds the best method for measuring structural factors such as socioeconomic status [94], as well as adherence [38]. Furthermore, these results highlight the lack of currently available research on social determinants of household support for ART adherence in the Middle East, the Caribbean, and other countries in South America, although the authors recognise that this may be the result of studies being accidentally missed if they were not captured by the language criteria for the review.

This review has several limitations. First, it was not possible to synthesise quantitative data using meta-analysis due to the considerable heterogeneity among the reviewed studies. Authors used a variety of measurements for social and structural determinants; adherence was measured by different methods in each of the studies, and the adherent/non-adherent percentage threshold varied between studies. Furthermore, the patients received different antiretroviral regimens, which is an important factor given that the complexity of the ART regimen may affect a patient’s adherence. This means effect sizes could not be calculated and the magnitude of the relationship could not be assessed. Second, although comprehensive in its scope, this review did not look at HIV testing, linkage to care, children’s transitions from child to adult ART care, pre- and post-exposure prophylaxis (PrEP/ PEP) studies, mental health, or alcohol or drug use. This meant that studies such as Conroy et al. (2019), which examined how gender and alcohol use intersected to affect household support for ART adherence, were not included [106]. We did not include interventions that focused on disclosure as a form of household support, as adherence is possible without disclosure; this did eliminate potentially interesting perspectives on structural determinants of disclosure. Third, reporting bias in the reviewed manuscripts may have limited identification of the myriad ways in which social and structural barriers intersect. For example, negative results (e.g., no effect or the opposite effect shown) may not have been developed further in analysis or reported in articles (either quantitative or qualitative). Indirect pathways and/or intersecting factors may be missed or possibly over-reported. Additionally, studies may have been missed because abstracts and titles did not refer to the ways the study examined structural factors affecting caregivers. Fourth, qualitative studies are not always found using conventional systematic review search strategies [107]. Although the team examined references for further studies, the possibility of having missed relevant studies cannot be excluded. 

Nevertheless, these findings have implications for public health promotion strategies, including for the design and development of family- and household-based interventions. Effective interventions should be multi-faceted, addressing behavioural, social and structural risk factors to maximise impact [22,33]. 

### Future Research

These results indicate important areas for future research to address:

(1) The majority of the studies reviewed focused on children and adolescents; further research on the social and structural determinants of household support for adherence among adults is needed to inform policy and intervention development.
(2) Important gaps in knowledge such as how disability and sexual orientation stigma affect support for ART adherence.
(3) The relationship between support given to household members, and the support they give to PLWH for adherence.
(4) A more nuanced approach is warranted in the analysis of caregiver education and socioeconomic status, the resulting impact on support, and how this affects ART adherence. Researchers should maintain awareness, for example, of how different measures of socioeconomic status may result in considerably different classifications of household wealth [108] and therefore lead to different conclusions on the relationship between poverty, support and adherence.
(5) The particular impact of gender, and religious and cultural health beliefs, on both household members and PLWH, and how this affects support for ART adherence.
(6) How social and structural determinants intersect, in order to identify particularly vulnerable groups.
(7) The effect of household support on ART adherence over time, and the impact of caregiver education, socioeconomic status, gender and decision-making power, which would better capture the changing influences on household support. Studies using path models could enable researchers to identify relationships between social determinants, household support, and ART adherence outcomes.

## 5. Conclusions

There is a clear need for more methodologically rigorous research on the social and structural determinants of household support for ART adherence, including using methods such as path analysis. Nevertheless, this review of qualitative, quantitative and mixed methods studies found that, according to the evidence currently available, social and structural factors affect the support given by household and family members to PLWH and that this may impact PLWH’s adherence to ART. Social support was crucial for household members to overcome the barriers they experienced in providing care and support to PLWH on ART. Intersecting social and structural determinants create particular challenges for household members who are older, female, with little education and low socioeconomic status. Household- and family-based adherence interventions should take account of, and, where possible, attempt to tackle, social and structural factors in order to have maximum impact on improving PLWH’s ART adherence. 

## Figures and Tables

**Table 1:** Search terms for web of science.

Database	Search Terms
Web of Science (TS = topic = title + abstract + key words) Filter: 2003–2019	#1 TS = (household* OR home* OR famil* OR couple* OR relationship* OR interpersonal) #2 TS = (adhere* OR complian*) #3 TS = (help OR support OR empower* OR care OR caring OR social support) #4 TS = (HIV OR ART OR ARV OR viral* OR CD4 OR pill count OR antiretroviral therapy OR antiretroviral treatment) #1 AND #2 AND #3 AND #4

**Table 2:** Assessed quality of studies categorised by each social/structural factor drawn from the review (and those referencing mitigation).

Social/Structural Factor	Studies of Good Quality	Studies of Fair Quality	Studies of Poor Quality
**Gender**	3 studies [46,61,78]	6 studies [58,62,64,66,75,81]	1 study [88]
**Education: literacy, language and knowledge**	2 studies [82,84]	8 studies [65,74,75,76,81,83,85,87]	3 studies [77,86,89]
**Religious & cultural health beliefs**	1 study [79]	8 studies [62,63,66,68,76,80,81,82]	2 studies [86,89]
**Socioeconomic status (SES)**	4 studies [46,61,79,82]	16 studies [59,60,62,63,64,65,67,68,69,71,73,76,80,81,85,87]	6 studies [70,72,77,86,88,89]
**Stigma**	2 studies [79,84]	17 studies [59,60,62,63,65,66,67,68,69,71,73,75,76,80,81,85,87]	4 studies [70,72,86,88]
**Health system factors**	2 studies [79,84]	7 studies [60,67,73,80,81,85,87]	2 studies [70,88]
**Caring for the carers: mitigating social and structural barriers**	2 studies [79,84]	11 studies [59,60,63,67,69,71,74,75,76,80,83]	2 studies [70,77]

## Supplementary Materials

The following are available online at https://www.mdpi.com/1660-4601/17/11/3808/s1, Supplementary 1: Search Terms for Seven Databases Searched; Supplementary 2: Tables Depicting Study Characteristics—Table S1: Qualitative Studies; Table S2: Quantitative studies; Table S3: Mixed methods studies.
