# Supplementary material for: Social and Structural Determinants of Household Support for ART Adherence in Low- and Middle-Income Countries: A Systematic Review
Source: Int J Environ Res Public Health. 2020 May 27;17(11):3808. doi: 10.3390/ijerph17113808 (PMC7312869; doi:10.3390/ijerph17113808)
Supplement: Supplementary file 1 [file ijerph-17-03808-s001.zip › Supplementary 1.pdf]

Supplementary 1: Search Terms for Seven Databases Searched.

Database	Search Terms
Web of Sciences<br>Filter: 2003-2019<br>*(TS = topic = title + abstract + key words)*	#1 TS = (household* OR home* OR famil* OR couple* OR relationship* OR interpersonal)<br>#2 TS = (adhere* OR complian*)<br>#3 TS = (help OR support OR empower* OR care OR caring OR social support)<br>#4 TS = (HIV OR ART OR ARV OR viral* OR CD4 OR pill count OR antiretroviral therapy OR antiretroviral treatment)<br>#1 AND #2 AND #3 AND #4
Cochrane/Evidence Based Reviews (OVID)	#1 (household* OR home* OR family OR families OR couple* OR relationship*).ti,ab,kf.<br>#2 exp family/ or exp interpersonal relations/<br>#3 (adhere* OR complian*).ti,ab,kf.<br>#4 exp patient compliance/ or medication adherence/<br>#5 (help OR support OR empower* OR care OR caring OR social support). ti,ab,kf.<br>#6 (HIV OR ART OR ARV OR viral* OR CD4 OR pill count OR antiretroviral therapy OR antiretroviral treatment).ti,ab,kf.<br>#7 exp hiv/<br>#8 exp anti-retroviral agents/ or exp anti-hiv agents/<br>#9 exp Anti-Retroviral Agents/ or Antiretroviral Therapy, Highly Active/<br>#10 1 or 2<br>#11 3 or 4<br>#12 6 or 7 or 8 or 9<br>#13 10 and 11 and 12 and 5<br>#14 13 and 2003:2019. (sa_year).
Medline<br>ti,ab,kf. = *title + abstract + key words*	#1 (household* OR home* OR family OR families OR couple* OR relationship*).ti,ab,kf.<br>#2 exp family/ or exp interpersonal relations/<br>#3 (adhere* OR complian*).ti,ab,kf.<br>#4 exp patient compliance/ or medication adherence/ or exp "treatment adherence and compliance"/<br>#5 (help OR support OR empower* OR care OR caring OR social support). ti,ab,kf.<br>#6 (HIV OR ART OR ARV OR viral* OR CD4 OR pill count OR antiretroviral therapy OR antiretroviral treatment).ti,ab,kf.<br>#7 exp hiv/<br>#8 exp anti-retroviral agents/ or exp anti-hiv agents/<br>#9 exp Anti-Retroviral Agents/ or Antiretroviral Therapy, Highly Active/<br>#10 1 or 2<br>#11 3 or 4<br>#12 6 or 7 or 8 or 9<br>#13 10 and 11 and 12 and 5<br>#14 13 and 2003:2019. (sa_year).
PubMed<br>Filters: Publication date from 2003/01/01 to 2019/12/31	#1 [Title/Abstract] household* OR home* OR famil* OR couple* OR relationship*<br>#2 [MeSH Terms] family and household<br>#3 [MeSH Terms] interpersonal relations<br>#4 [Title/Abstract] adhere* OR complian*<br>#5 [MeSH Terms] patient compliance<br>#6 [MeSH Terms] medication adherence<br>#7 [MeSH Terms] "treatment adherence and compliance"<br>#8 [Title/Abstract] help OR support OR empower* OR care OR caring OR social support<br>#9 [Title/Abstract] HIV OR ART OR ARV OR viral* OR CD4 OR pill count OR antiretroviral therapy OR antiretroviral treatment<br>#10 [MeSH Terms] anti-retroviral agents OR<br>#11 [MeSH Terms] anti-hiv agents OR<br>#12[MeSH Terms] Anti-Retroviral Agents<br>#13 [MeSH Terms] Antiretroviral Therapy, Highly Active<br>#14 #1 OR #2 OR #3<br>#15 #4 OR #5 OR #6 OR #7<br>#16 #9 OR #10 OR #11 OR #12 OR #13<br>#17 #14 AND #15 AND #8 AND #16
EMBASE<br>Filter: 2003–2019	household* OR home* OR famil* OR couple* OR relationship* OR interpersonal<br>AND

	adhere* OR complian*<br>AND<br>help OR support OR empower* OR care OR caring OR social support<br>AND<br>HIV OR ART OR ARV OR viral* OR CD4 OR pill count OR antiretroviral therapy OR antiretroviral treatment
CINAHL<br>(EBSCO)<br>Filters ticked:<br>peer-reviewed<br>Jan. 2003–Dec. 2019<br>'suggest subject terms' not ticked	(household* OR home* OR famil* OR couple* OR relationship*) OR MH "Interpersonal Relations+" OR MM "Interpersonal Relationships (Omaha)" OR (MH "Family")<br>AND(adhere* OR complian*) OR MM "Medication Compliance" OR MH "Patient Compliance+" OR MM "Compliance with Medication Regimen (Saba CCC)"<br><br>AND<br>(help OR support OR empower* OR care OR caring OR social support) OR (MH "Support, Psychosocial+")<br><br>AND<br>(HIV OR ART OR ARV OR viral* OR CD4 OR pill count OR antiretroviral therapy OR antiretroviral treatment) OR (MM "Antiretroviral Therapy, Highly Active") OR (MH "Anti-Retroviral Agents+") OR (MM "HIV-AIDS Nursing") OR (MH "Human Immunodeficiency Virus+")
Psych-ARTICLES<br>(ProQuest)<br>Filter: Date: From 01 January 2003 to 31 December 2019	#1 TIABSU(household* OR home* OR famil* OR couple* OR relationship* OR interpersonal)<br>#2 TIABSU(adhere* OR complian*)<br>#3 TIABSU(help OR support OR empower* OR care OR caring OR social support)<br>#4 TIABSU(HIV OR ART OR ARV OR viral* OR CD4 OR pill count OR antiretroviral therapy OR antiretroviral treatment)<br>#1 AND #2 AND #3 AND #4
