# Supplementary material for: Social and Structural Determinants of Household Support for ART Adherence in Low- and Middle-Income Countries: A Systematic Review
Source: Int J Environ Res Public Health. 2020 May 27;17(11):3808. doi: 10.3390/ijerph17113808 (PMC7312869; doi:10.3390/ijerph17113808)
Supplement: Supplementary file 1 [file ijerph-17-03808-s001.zip › Supplementary 2.pdf]

Supplementary 2: Tables Depicting Study Characteristics

Tables S1: Depicting Study Characteristics

Author and year of publication	Location of study	Study Design (and analytical method if stated)	The household/family member giving support	The household/ family member receiving support for ART adherence	Sample description	Structural social determinants	Type of support given by a household member to patient: instrumental, emotional or informational	Effect of structural/social determinants of household support for ART adherence	ART adherence
**Bhagwanjee et al., 2013**<br><br>**(58)**	Carlton, South Africa.	Semi-structured couple interviews. Inductive emergent thematic analytic method.	Seroconcordant couples - couple (partner) support.	Seroconcordant couples - couple (partner) support.	12 seroconcordant heterosexual couples.	Gender norms	Instrumental.<br><br>Emotional.	Gender norms affected whether instrumental or emotional support was given.	Self-report; the majority of the sample reported sub-optimal adherence to their ARV medication.
**Bikaako-Kajura et al., 2006**<br><br>**(59)**	Kampala, Uganda.	In-depth interviews, plus short demographic questionnaire.	Caregivers: a person who lives with the child, participates in the child's daily care and is the most knowledgeable about the child's health and adherence. Either biological parents or guardians perceived by the child as a replacement for deceased parents.	Children living with HIV.	42 caregivers and 42 HIV+ children, 42 caregivers (median age of 37) and 42 HIV+ children (aged 5-17 years old).	Socioeconomic status<br>Stigma	Instrumental.	Socioeconomic status: poverty affected transport and food security.<br><br>Stigma affected disclosure (disclosure affected adherence).	Self-report: 12 (29%) of the children and their caregivers reported excellent adherence (never missed any dose), 17 (40%) good adherence (occasionally missed doses) and 13 (31%) poor adherence (frequently missed doses). Individual and contextual factors had a stronger influence on adherence experiences than medication type.
**Busza et al., 2014**<br><br>**(60)**	Harare, Zimbabwe.	Semi-structured interviews. Framework analysis.	Caregivers: family members (mothers, fathers, aunts, grandmother, cousins).	Children living with HIV aged 6-15.	15 primary caregivers, 9 key informants from 5 community-based organsations providing adherence support or related services.	Socioeconomic status<br>Stigma<br>Health system	Instrumental.<br><br>Emotional: positive living more broadly (mitigation of stigma, relationships at school, nutrition, psychosocial care, etc).	Socioeconomic status: opportunity cost of attending appointments; food insecurity mentioned but did not appear to affect adherence.<br>Stigma in the community;<br>Health system: relationship with health staff, drug shortages; health system capacity.	Described in general terms.
**Conroy et al., 2017**<br><br>**(61)**	Vulindlela, Kwa-Zulu-Natal, South Africa.	In-depth semi-structured interviews.	Supporting partner (couples)	Adult PLWH.	24 couples (48 individuals). Mean age 35.5 years (range 22-51).	Socioeconomic status<br>Gender norms	All three: instrumental, informational and emotional support.	Normative gender roles led to conflict (and IPV) when these roles were not fulfilled. Relationship conflict could interfere with adherence: gender roles permitted violence, which had a negative effect on ART adherence as men either took away ART medication or the woman did not ask for help with adherence for fear of violence.	The majority of couples were seroconcordant HIV-positive (92%) and both on ART (63%). Of the 33 partners on ART, the average time on ART was 28.7 months (range: 2–158) and 81.8% reported perfect adherence to ART in the past 30 days.
**Conroy et al., 2018**<br><br>**(46)**	Zomba, Malawi	80 in-depth interviews with 25 couples. Innovative analysis approach.	Supporting partner (couples)	Adult PLWH.	25 married heterosexual couples with at least one HIV+ partner.	Socioeconomic status<br>Gender norms	Instrumental: bringing pills, reminders and collecting pills at the clinic. Men financially supported the household.<br><br>Exchange: some couples noted that ART led to men terminating their extramarital partnerships in exchange for love and support.	Socioeconomic status: impacts marital strain and creates stress, which affects communal coping for ART adherence.<br><br>Gender: Women have less power in relationships - their husbands' HIV status enabled women to leverage decision making power within the relationship, which led to better support.	Self-report.
**Demmer et al., 2011**<br><br>**(62)**	South Africa.	In-depth interviews. Thematic analysis.	Mothers or a female relative entrusted with care of the child.	Children.	13 mothers of children living with HIV and 12 professional caregivers.	Stigma<br>Socioeconomic status<br>Gender norms<br>Health beliefs	Instrumental: nutrition, clinic visits, taking medication on time.<br><br>Emotional: helping child cope with loss of mother.	Stigma: caregivers did not disclose the status of their children; caregivers did not therefore have sufficient financial or emotional support themselves from family, so they coud not give adequate care to their children.<br>Gender norms: placed women as caregivers.<br>Socioeconomic status: caregivers had difficulty finding money for transport costs, taking time off work, long waits to be seen and no money for food.<br>Health beliefs: belief in traditional medicines and witchcraft meant that caregivers did not ensure effective adherence.	Discussed in general terms.<br><br>Not all the children were on ART: some had stopped for various reasons (e.g.clinic shortages), and some of the mothers were on a waiting list.

Author and year of publication	Location of study	Study Design (and analytical method if stated)	The household/family member giving support	The household/ family member receiving support for ART adherence	Sample description	Structural social determinants	Type of support given by a household member to patient: instrumental, emotional or informational	Effect of structural/social determinants of household support for ART adherence	ART adherence
**Fetzer et al., 2011**<br><br>**(63)**	Kinshasha, DRC.	40 interviews. Qualitative content analysis.	Caregivers: family and non-relatives. The primary caregiver was defined as the adult who primarily serves to assist the child with taking ART	Children living with HIV, 8-17 yrs old.	20 sets of children between 8 and 17 years of age and their respective primary caregivers.	Socioeconomic status<br>Stigma<br>Religious beliefs	Instrumental: motivating children to take their doses, by inducement with gifts, or fear. Strategies for being reminded to take medication: caregivers would set phone or clock alarms at appropriate times. Keeping logs as a record of completed doses was also mentioned.	Socioeconomic status: Lack of nutritional support cited by both children and caregivers for missed doses.<br><br>Stigma: meant concealment of status from family and children, therefore caregivers received no help, and children resisted taking medication.<br><br>Religion gave caregivers courage to continue supporting their children.	Only 6 caregiver-child (33%) sets concurred regarding the most recently missed ART dose. The majority of ART regimens consisted of standard first line pediatric treatment zidovudine or stavudine plus lamivudine plus nevirapine or efavirenz. Seven of the children were on alternate or second-line pediatric regimens. Poor adherence noted by clinic staff for 19 of the children. Reasons noted included lack of assistance in the home, repeatedly missing clinic appointments, stated mistrust in the medications.
**Kohli et al., 2012**<br><br>**(64)**	Pune, Maharashtra, India.	8 focus group discussions and 44 in-depth interviews. Thematic analysis.	Family members, identified by PLWH.	Adult PLWH.	PLWH (20) and their family members (24), key informants for FGDs (88).	Socioeconomic status<br>Gender norms	Emotional support.<br><br>Instrumental: nursing and financial support to PLHIV.	Socioeconomic status: money mostly spent on food, leaving little else. Food prices are high.<br>Gender: women are primary caregivers. Men give money and women are responsible for keeping men alive. Unconditional support expected because men need to earn. Therefore they receive care even when resources are limited. Women receive little/ no care, limited support.	Discussed in general terms.
**Mafune et al., 2017**<br><br>**(65)**	Mutale Municipality, Vhembe District, Limpopo Province, South Africa.	In-depth, individual, unstructured interview. Tesch's open-coding method.	Family members	Children living with HIV, on ART, aged 0 to 15 years.	16 caregivers 18 years of age and older, caring for children on ART aged 0 to 15 years.	Socioeconomic status<br>Stigma education and literacy<br>Health knowledge on disability	Instrumental: support with taking medicine, financial support, caring for children, attending appointments, preparing food.	Socioeconomic status: access to the clinic and appointments for obtaining ARVs is difficult.<br>Education and literacy levels hindered proper treatment management, disclosure and non-disclosure challenges.<br>Disability meant caregivers didn't know how best to support their learning-impeded child.<br>Stigma: no disclosure, so children had no idea why they were taking drugs, taken in secret.	Self-report: caregivers reported frequent dose missing/being late taking doses.
**Martinez 2018**<br><br>**(66)**	Guayaquil, Ecuador.	59 in-depth interviews. Grounded theory. Ethnographic research	Family and health professionals (doctors, nurses, psychologists, etc.)	Adults living with HIV.	17 adults living with HIV. 10 doctors. 16 other professionals in the hospital (psychologists, pharmacist, social workers, nurses).	Gender norms<br>stigma<br>Health beliefs	Instrumental: support with taking medication (women), financial support (men), picking up medication from the hospital (both).<br><br>Emotional support (women).	Gender: men give money, women give time.<br>Stigma and gender norms intersect to affect amount of care given and to whom, based on ideas of culpability and gender norms: the higher the culpability, the lower the amount and quality of care given. Culpability refers to mode of acquisition from 'best' to 'worst': i) blood transfer, job exposure, perinatal; ii) married woman who has sex with husband; iii) heterosexual man who has extramarital sexual relations with women; v) single woman with no stable relationship; v) Men who have Sex with Men (MSM).	Discussed in general terms.
**Nasuuna et al., 2019**<br><br>**(67)**	Uganda.	5 focus group discussions. Thematic analysis, inductive approach.	Primary caregivers of children: biological parents or guardians	Children, non-virally suppressed.	37 caregivers, age 15 to over 50.	Socioeconomic status<br>Stigma<br>Health system	Instrumental: reminding to take medication, taking to the facility, ensuring food is available,<br><br>Informational: discussing ART with doctors and counsellors.	Socioeconomic status: caregiver has to work -cannot support child as not present. Lack of food and infrequent meals prevented children from taking drugs.<br>Stigma: caregiver has no support from family who have abandoned them and experiences burnout - gives less effective support, affecting child's adherence.<br>Health system: long queues and early closure mean obtaining ARV's is difficult.	Discussed in general terms.

Author and year of publication	Location of study	Study Design (and analytical method if stated)	The household/family member giving support	The household/ family member receiving support for ART adherence	Sample description	Structural social determinants	Type of support given by a household member to patient: instrumental, emotional or informational	Effect of structural/social determinants of household support for ART adherence	ART adherence
**Nestadt et al., 2018**<br><br>**(68)**	Thailand.	2 focus group discussions and 6 in-depth key informant interviews. Framework analysis.	Primary caregivers of children.	Adolescents.	10 children 13–16 years old and 8 adults.	Stigma<br>Socioeconomic status<br>Social norms around communication.	Instrumental: medication reminders.<br><br>Emotional: motivation to take ART drugs.	Stigma: affected disclosure which affected adherence<br><br>Socioeconomic status: caregivers are away from home earning an income, so are not at home to help children with medication.<br><br>Social norms around family hierarchy and communication created intergenerational conflict.	Discussed in general terms.
**Olds et al., 2014**<br><br>**(69)**	Uganda.	Interviews. Inductive content analysis. Nested qualitative study with participants of a longitudinal study of real-time ART monitoring.	Primary caregivers of children.	Children, (median age of 7 years (IQR 5–8)).	35 caregivers, median age was 41 years (interquartile range [IQR] 33–48), of Children living with HIV. Sampled from two groups of pediatric participants (viral suppression or not virally suppresed: Viral suppression as HIV RNA <1,000 copies/ml).	Socioeconomic status<br>Stigma	Instrumental: food provision, transport to the clinic, taking out loans to ensure financial support for transport, ensuring timetable of care, collecting tablets, reminders for taking pills.<br><br>Emotional: love and esteem, prayers.	Socioeconomic status: competing priorities for household expenditure, affects drug adherence.<br>Stigma: prevents caregivers from reciving social support from family as it prevents disclosure.<br>Social support for caregiver enables them to overcome barriers. Caregivers in both groups accessed social support; however, greater level and availability of private instrumental support among caregivers ofvirally suppressed children, more apparent in discussions. These caregivers appeared to have more options for private instrumental support within and outside of the household.	Caregivers: 60 % were infected with HIV, 34 % were receiving ARVs. Their children's median duration of ARV therapy was 5 years (IQR 5–6) and 97 % were on first line ARV regimens.<br><br>Adherence monitored over a 6-month period with a medication container (Wisepill), which transmits a cellular signal upon opening.<br><br>Seventeen participants had HIV RNA ≥1,000 copies/ml, and 18 participants had HIV RNA < 1,000 copies/ml. Median adherence of 93 % (IQR 89–95 %) and 93 % (IQR 89–96 %), respectively.
**Paranthaman et al., 2009**<br><br>**(70)**	South India.	In-depth interviews. Framework analysis.	Primary caregivers of children: mothers, fathers, step-parents, grandparents.	Children up to 12 years.	14 caregivers responsible for routinely administering ART to the child.	Stigma<br>Socioeconomic status<br>Health system	Instrumental: collecting drugs, paying for drugs.<br><br>Emotional: encouraging to take medication.<br>Negative emotional: forcing to take medication.	Stigma: no disclosure was made, medications were forced down with beatings. Affected use of services; caregivers travelled long distances to use different clinics which cost money and therefore affected what ART could be bought.<br>Socioeconomic status: can't pay for ART at private clinic.<br>Health system: no trust in government ART quality, so don't take the free drugs, and can't always afford the expensive ARVs.	If the child and caregiver had not missed any dose of medication in the last three days, they were considered to be adherent.<br>Caregivers stating good adherence (out of 14): 8, non-adherent: 2.<br>Caregivers reporting children's adherence (out of 14): Adherent 12, non-adherent 2.
**Petersen et al., 2010**<br><br>**(71)**	Durban, KwaZulu-Natal, South Africa.	In-depth qualitative interviews. Thematic coding.	Primary caregivers of children: biological mother, grandmother, aunt, uncle, children's orphanage caregiver.	Adolescents.	25 adolescents living with HIV, 15 caregivers.	Socioeconomic status<br>Stigma.	Instrumental: financial support<br><br>Emotional support.	Socioeconomic status: lack of money for food/transport to get drugs.<br>Stigma: affected caregiver's disclosure to wider community and family, which affected adherence support because the caregiver had no support with burden of care. Caregivers experiencing difficulty coping with caring for a child living with HIV were either on their own with their children or in an unsupportive family arrangement.	Discussed in general terms.
**Punpanich et al., 2008**<br><br>**(72)**	Bangkok, Thailand.	In-depth interviews using a semi-structured interview guide.	Primary caregivers of children with HIV/AIDS: 3 fathers, 11 mothers, 16 relatives, 1 foster parent.	Children.	34 children age 8–16 years and 35 primary caretakers.	Socioeconomic status (linked to age)<br>Stigma.	Instrumental: ensuring regimen adhered to, picking up drugs, storing drugs.	Socioeconomic status: treatment is paid for until the child is age 18, but not afterwards. How will grandparents afford it, epsecially with limited economic opportunity in old age?<br>Stigma: can't find someone they trust to care for child in their absence. No disclosure to families. Adherence is difficult when others are present.	Discussed in general terms.

Author and year of publication	Location of study	Study Design (and analytical method if stated)	The household/family member giving support	The household/ family member receiving support for ART adherence	Sample description	Structural social determinants	Type of support given by a household member to patient: instrumental, emotional or informational	Effect of structural/social determinants of household support for ART adherence	ART adherence
**Purchase et al., 2016**<br><br>**(73)**	South Africa,Western Cape.	Semi-structured interviews with staff, caregivers and patients.	Primary caregivers of children, nurses and community voulnteers: immediate relatives, mother, father, grandmother, and non-immediate relatives.	Children.	21 caregivers and 4 children (aged 11–13 years).	Socioeconomic status<br>Stigma<br>Health system	Instrumental: administer tablets, pick up tablets, take children to appointments.	Socioeconomic status: cost of transport doesn't affect caregivers because they live close to the clinic.<br>Stigma: doses are kept secret, can't be seen to take medication. Affects disclosure.<br>Health system: availability of drugs, labels and formats affected understanding and trust.	Children with a viral load of >1000 copies/ml were purposively sampled.
**Remien et al., 2013**<br><br>**(74)**	Cape Town, South-Africa.	Feedback from interviews and focus group discussions regarding the multi-media based ART adherence intervention adapted from the SMART intervention, for South Africa.	Patient-selected treatment support partner: friends, family, boyfriend, spouse.	Adults with reported poor adherence.	33 patients, 33 treatment-partners, two lay counselors, six clinic providers.	Education and literacy.	Instrumental: remembering medication, accompany to clinic.<br><br>Emotional and informational problem-solving support - helping the patient to identify barriers to adherence and brainstorm solutions.	Level of education and literacy affects knowledge of HIV and ability to engage with materials explaining the virus. The intervention explicitly dealt with issues around low education levels and illiteracy by designing the intervention with this in mind. The intervention therefore uses multimedia, to help family members and patients with low literacy levels to understand the material.	Patients with low adherence were selected on the basis of pharmacy records and/or clinician assesment.
**Scott et al., 2014**<br><br>**(75)**	Manicaland, eastern Zimbabwe.	67 interviews and 8 focus group discussions. Over 100 hours of ethnographic observation. Thematic network analysis.	Family of PLWHA: family included children, grandparents, parents, extended family networks, carers of children on ART, partners.	Adults and children.	25 health care workers, 62 adults on ART, 40 carers of children on ART.	Gender norms<br>Stigma<br>Socioeconomic status<br>Literacy	Instrumental: financial/food, taking medication.<br>Negative instrumental: male partners undermine treatment by withholding financial resources, refusing to let wives take ARV, stealing medication, stopping wives from attending clinic.<br><br>Negative emotional: men prevented women from attending support group meetings.	Women are financially dependent on husbands and were unable to access treatment if husbands did not financially support them. Men felt that HIV diminished their masculinity, feminised them.<br>Literacy: children had higher literacy levels than parents and could therefore help with ART adherence by reminding them. Younger children also worked and contributed money to the household.<br>Food interventions: important because these support older people in supporting orphans, and men to reassert masculinity.	Discussed in general terms.
**Shaibu, 2015**<br><br>**(76)**	Ramotsa village, Botswana.	12 semi-structured interviews. Grounded theory.	Grandmothers of HIV-positive grandchildren.	Grandchildren with HIV	12 grandmothers (between 60-80 years old, mean = 68).	Education and literacy<br>Stigma<br>Cultural health beliefs<br>Socioeconomic status	Instrumental: support with taking medication, going to clinic, picking up medication.	Education and illiteracy: grandmothers couldn't read appointment cards and relied on children to do it or otherwise forget when to go to the clinic. Literate grandchildren gave support to grandparents.<br>Stigma and cultural health beliefs: illness is associated with witchcraft, which means there is no disclosure to community or child, and secrecy in taking meds. Difficulty of talking about sex because of social norms means that no disclosure is made in case have to discuss sex.<br>Socioeconomic status: Food parcels for children living with HIV is met with scorn by the community. No bus money, don't go to the clinic.	Discussed in general terms.
**Skovdal et al.a., 2011**<br><br>**(77)**	Manicaland, eastern Zimbabwe, 3 rural communities.	26 in-depth interviews, 1 FGD. Social constructionist perspective, thematic network analysis.	Grandparents. Grandparents also receive support from grandchildren.	Children, for ART medication, but they also give support to their grandparents.	8 grandparents over 50 (mean age 61), 25 nurses.	SES (and age), literacy and education.	Instrumental: getting pills from pharmacy, taking pills (remembering/dosing), food for treatment, taking the patient to the clinic.	SES: age limits economic ability to help ART adherence, can't afford food or transport, children have to contribute to the household and ill children are forced to work. Age-related physical frailty means they can't carry small children to the clinic. Older caregivers more likely to get sick and be periodically immobile, preventing the child from attending clinic and pick up ARV supplies. Poor memory: can't remember appointments or regime.<br>Literacy: can't read or write so can't comprehend regimen. If the child is still young, they cannot assist in keeping notes for their treatment schedule.	Self-reported, discussed in general terms.

Author and year of publication	Location of study	Study Design (and analytical method if stated)	The household/family member giving support	The household/ family member receiving support for ART adherence	Sample description	Structural social determinants	Type of support given by a household member to patient: instrumental, emotional or informational	Effect of structural/social determinants of household support for ART adherence	ART adherence
**Skovdal et al.b., 2011**<br><br>**(78)**	Zimbabwe.	37 individual interviews 5 FGDs. Thematic network analysis.	Men (husbands).	Women (wives) living with HIV.	32 women and 21 men.	Gender norms.	Negative instrumental: men prevent women from going to the hospital to get drugs, or threaten divorce if she uses ARV's, or they steal her drugs.<br><br>Negative emotional: men insist on no condom use, so threaten women with re-infection.	Gender norms: HIV threatens what it means to be a man and they fear being exposed as living with HIV themselves. Men therefore prevent women from going to the hospital to get drugs, or threaten divorce if she uses ARV, or they steal her drugs, or insist on no condom use, so threaten with re-infection.	Discussed in general terms.
**Vreeman et al., 2009**<br><br>**(79)**	Western Kenya.	10 FGDs and 35 individual interviews. Grounded theory.	Parents and care-givers.	Children on ART.	120 parents and caregivers, children of ages 0-14 (mean 6.8).	Socioeconomic status<br>Stigma<br>Cultural health<br>Health system	Instrumental: collecting medication, taking medication, remembering medication, food with medication.	Socioeconomic status: no food and no money for transport to the clinic, drugs are not given and not picked up. Rural location compounds this issue.<br>Stigma: affects caregiver disclosure to other adults and children, so doses are delayed when visitors are present. No support from community means children's adherence unsupported when caregiver is at work.<br>Cultural beliefs: different levels of support given by community.<br>Health system: punitive methods of communication mean that non-adherence is not discussed with doctor.	Discussed in general terms.
**Ware et al., 2008**<br><br>**(80)**	Jos, Nigeria; Dar es Salaam, Tanzania; Mbarara, Uganda.	Ethnographic design: 414 in-person interviews and 136 field observations of clinic activities.	Treatment support partner: family or friends. "As family members or friends, treatment supporters often live in the same or a nearby household".	Adult family members or friends.	158 patients, 45 treatment partners and 49 health care providers.	Socioeconomic status<br>Health system	Instrumental: financial support (and or food and transport), borrowing and begging on patients behalf, reminders, observing taking medication, practical care like cooking.<br><br>Emotional: expectations of adherence (which motivates patients to adhere), encouragement.<br><br>Emotional and informational: destigmatising HIV by socialising with patients and challenging health myths by sharing food and utensils, taking responsibility for someone's health in general.	Socioeconomic status: financial support, in the form of borrowing from friends to give to the patient meant that the patient could attend the clinic and obtain their drugs. Caregivers draw on social capital to support patient, who repays with adherence.<br><br>Economic barriers to ART adherence are mediated and overcome by social support that often takes the form of financial support for transportation and food and reminders to take medication.	Information on adherence collected through the 3-d self-report instrument of the AIDS Clinical Trials Group . Adherence subsequently discussed in general terms.
**Yeap et al., 2010**<br><br>**(81)**	Gauteng Province, South Africa.	In-depth interviews.	Caregivers: family members (mother, aunt, father, grandmother).	Children.	21 caregivers, 21 clinic staff members, 3 lead members of staff. Caregivers 21-65 years, median of 33 years.	Socioeconomic status<br>Gender norms<br>Stigma<br>Health system<br>Cultural health beliefs	Instrumental: taking medication, taking to the clinic.	Socioeconomic status: no money for transport to the clinic, or for drugs for opportunistic infections.<br>Gender and Stigma: don't take children to the clinic for fear of upsetting the father. Mother's status is inferred from the child's.<br>Health system: long waiting times and rude staff, so are reluctant to return for fear of reproach.<br>Health beliefs: believing that knowing a child's status will cause the illness to progress faster.	Discussed in general terms.

Supplementary 2: Tables Depicting Study Characteristics

Table S2. Quantitative studies.

Author and date of publication	Location of study	Study Design	The household / family member giving support	The household / family member receiving support for ART adherence	Sample description	Missing/lost to follow up (LTFU)?	Structural social determinants	Measurement of social determinants	Type of support given by a household member to patient: instrumental, emotional or informational	Effect of structural/social determinants of household support for ART adherence	Adherence: pill count	Adherence: viral suppression	Other items
**Davies et al., 2008**<br><br>**(82)**	South Africa.	Cohort study.	Primary carer of child: assumed to be the parent (parents gave consent) but not explicitly defined.	PLWHA children.	122 children on ART, median age of 37 months (range 16-61); 87/98 caregivers completed the questionnaire.	13 deceased, 11 LTFU.	Socioeconomic status<br>Education<br>Health beliefs	Caregiver secondary education: Yes / No / Unknown.<br>Caregiver employed:<br>Yes / No / Unknown .<br>Caregiver/child receives social grant: Yes / No / Unknown.<br>Formal housing:<br>Yes / No / Unknown.<br>Access to water and electricity:<br>Yes / No .<br>Access to working refrigerator:<br>Yes / No.	Instrumental: reminding and giving medication. Caregiver reported difficulty with giving medication associated with annual average MR adherence < 90% (OR: 3.07, 0.91-10.38, p=0.06).	Secondary education of caregivers as predictor of MR: OR: 4.49 (1.10-18.24).<br>Access to water and electricity as predictor of MR: OR: 2.65 (0.93-7.55).	91 (79%) achieved annual average MR adherence ≥ 90%.	MR and viral suppresion: OR=5.5 (0.8-35.6), controlled for disease severity.	Caregiver health beliefs did not influence adherence by any measure.
**Knodel et al., 2011**<br><br>**(83)**	Cambodia and Thailand.	Cross-sectional.	Older family members; parent or grandparent.	Adult ART patient who is nevertheless the child or grandchild of the caregiver.	340 adult ART recipients in Cambodia and 912 recipients in Thailand; the second round included 108 older people in each country who were involved with an ART recipient.	None.	Education and health literacy	Education in years.	Instrumental: preparing medication, accompanying patients to clinic reminding them to resupply. Cambodian ART recipients with a living parent were noticeably more likely to report parental assistance in these matters than their Thai counterpoints. Still over half (54%) of Thai recipients with a living parent and over 70% of those co-resident with a parent indicated they had received parental assistance in at least one of these three ways.	Knowledge of ART is needed for effective assistance for adherence. Education was associated with knowledge in the Cambodian sample (p<0.05) but not in the Thai sample (p>0.05).<br><br>In both countries, those with better ART knowledge more likely to report that they reminded their adult child or relative to take drugs: in Cambodia, 100% of those scoring above the median reminded the patient to take ARVs compared to 47% of those with knowledge scores below. In Thailand, 76% whose knowledge score was above the median compared to 60% whose score was below gave reminders.	Patients were engaged in care: adherence was not measured as all cases were engaged in care and the survey focus was on what supported them to adhere.	n/a	The household effect: in both settings and when controlled for co-residence, large proportions of co-residing family members are said to help remind ART recipients to take medication.
**Muller et al., 2011**<br><br>**(84)**	Cape Town, South Africa.	Cross-sectional.	Caregivers who are responsible for medication administration. 80% were biological parents. Unclear status of the remaining 20%.	Children no older than 10 years.	57 caregiver-child dyads. The children had to be 10 years or younger (mean age 51 months SD25.6), and the caregivers had to be responsible for medicine administration.	Original sample of 78 dyads; missing data on viral suppression or adherence meant 57 were included in the analysis.	Socioeconomic status<br>Education<br>Stigma<br>Health system	Years of education.<br>Monthly income.<br>Home language.<br>Existence of a tap at home.<br>Existence of electricity at home.<br>Possession of a watch or cell phone.<br>Living in informal housing.<br>Transport costs prevent clinic attendance.	Instrumental: caregivers were responsible for administration of medicines. Also provided financial support.	Confidence in speaking English with the doctor significantly correlated with viral suppression: (r=0.34, p<0.01) (not education).<br>Stigma: associated with lower viral suppression - presumably because stigma meant greater levels of secrecy for the caregiver to navigate.<br>Education and poverty were not significantly associated with adherence.	Adherence electronically monitored over three months. Mean adherence was 81%, but only 39% had adherence levels of 95% or above.	Viral load of <50 copies defined as VS. 67% of children achieved virologic suppression. Association between continuous adherence and viral load (r=-0.47, p<0.001).	A factor model with child's health status, caregiver's language skills, caregiver's disclosure and perceived stigmatisation could explain 95% of the variation in viral suppression.
**Polisset et al., 2008**<br><br>**(85)**	Lome, Togo.	Cross-sectional.	Primary caregivers of children: biological parent, adoptive parent, foster parent or other relative who looks after a child including responsibility for ART administration.	Children 15 years of age or less, receiving ART.	74 children (0-15 years), (median age 6 years) and 42 caregivers.The median age of the children was 6 years (IQR 4-9). The median age of the caregivers was 37 years old (IQR = 32–45). 57% of caregivers declared they were HIV+. 22 were receiving ART in the same centre as the child.	Of 83 children at the HIV centre receiving ART, nine not enrolled in study because had no caregiver.	Socioeconomic status<br>Health system factors<br>Education<br>Stigma	Caregiver's high education level (secondary school or above): yes/no.<br>Caregiver's reported knowledge of HIV:<br>yes/no.<br>Living conditions:<br>Community setting<br>Individual house	Instrumental: responsibility for administering the drug regimen.<br>Paying for drugs: eleven caregivers reported paying for ART.	Socioeconomic status: caregivers stated they need financial support to care for their HIV+ children.<br>Education and knowledge of HIV/ART: Not associated with adherence: caregiver educational level OR education secondary or above 0.8 [0.3–2.1], disclosure of child's HIV sero-positive status, and caregiver having basic knowledge of HIV 0.5 [0.1–1.3].<br>In multivariate analysis, caregivers' reporting no difficulty with ARV administration remained independently associated with the reported child's non-adherence: OR 0.09 [0.009–0.1]*P<.05.<br>Health system: stock-outs.<br>Stigma: Non-disclosure.	Caregivers' report of no antiretroviral drug doses missed either in the past 4 days or in the month before the interview. 42% of caregivers declared perfect adherence.	n/a	Univariate analyses: non-adherence assoc. with: living in an individual setting (vs. compound with enlarged family), caregiver other than biological parent, caregiver not declaring HIV-status, not participating in support groups and having perceived difficulty of antiretroviral (ARV) administration.<br>Multivariate analysis: female gender (adjusted odds ratio [aOR]: 3.8; CI: 1.3–12.6), living in an individual setting (aOR: 6.0; CI: 1.6–21.8), receiving other than NNRTI-based regimen (aOR: 3.9; CI: 1.1–13.4) and caregivers' perceived difficulty of ARV administration (aOR: 10.6; CI: 1.0–111.1)

Supplementary 2: Tables Depicting Study Characteristics

Table S3. Mixed methods studies.

Author and date of publication	Location of study	Study Design	The household/ family member giving support	The household/ family member receiving support for ART adherence	Sample description	Missing/lost to follow up (LTFU)?	Structural social determinants	Measurement of social determinants	Type of support given by a household member to patient: instrumental, emotional or informational	Effect of structural/social determinants of household support for ART adherence	ART adherence doses missed	ART adherence viral suppresion
**Fredriksen-Goldsen et al., 2011 (86)**	Beijing, China.	Cross-sectional survey and individual semistructured interviews.	Family member or friend providing informal assistance and available to support the person living with HIV.	Adults living with HIV.	Qualitative: patients living with HIV (n = 10) and their family caregivers (n = 10).<br>Quantitative: 70 patients initiating ART and an additional 50 ART-experienced patients who were not involved in the intervention trial.	7 did not respond to the adherence questions, reducing the sample size (quan) to 113.	Socioeconomic status Education Stigma	Education:<br>High school or less<br>Higher than high school.<br><br>Employment:<br>Employed Yes/No<br><br>Income:<br>Household income<br>< $2,000<br>$2,000 +<br><br>Residence:<br>Urban/Rural.	Instrumental, informational and emotional<br>Instrumental support consists of lending money, help with medication instructions and regimens, reminders to take pills and help getting medication.<br>Informational support: sharing knowledge.<br>Emotional support: motivating the patient. Patients identified the support of their caregivers as enhancing their will to survive, which, in turn, promoted their ART adherence.	Socioeconomic status: Caregivers used their own salaries for patient ART, which caused them worry and economic hardship. Household income was not related to support or adherence.<br>Education: Limited education meant it was difficult to understand ART regimens.<br>Larger household size meant support was more likely.<br>An urban residence promoted adherence.<br>Family support was found to help adherence after controlling for other covariates. Patients who were married or partnered (v2 = 16.40), p < .001, with larger household size were [t(111)= -2.17], p < 0.05, were more likely to receive family caregiving support. Those who were adherent were more likely to have a caregiver who helped them with their HIV care (v2 = 5.18), p < 0.05.	Self-report measures	n/a
**Tran et al., 2016 (87)**	Hannoi and provinces, Ho Chi Min and surroundings, Vietnam.	Cross-sectional (questionnaire), and 8 focus-group discussions.	Parents and other primary and non-primary caregivers: 58% were biological parents, the rest were 'other'.	Children.	Qualitative: 53 caregivers.<br>Quantitative: 209 caregivers.	n/a	Stigma<br>Socioeconomic status<br>Health system	Job:<br>Employed<br>Unemployed<br><br>Education:<br>Under high school<br>High school or above<br><br>Family residence:<br>Urban area<br>Rural area	Instrumental<br>Instrumental support consisted of getting meds from the clinic, help taking meds, financial support (food/clothes/school), going to clinic.	Socioeconomic status: rural area meant higher cost of transport, which compounded high levels of poverty. Patients could not go to the clinic to get drugs which impeded adherence.<br>Health system: fixed schedules with clinic visits during school hours and drug pick-ups requiring the presence of the child.<br>Stigma: prevented wider disclosure, which meant secondary caregivers could not maintain adherence when the primary caregiver was at work.	Adherence not measured, discussed qualitatively.	n/a
**Weigel et al., 2009 (88)**	Lilongwe, Malawi.	Cross-sectional survey and 4 focus group discussions (critical incident narrative).	Caregivers: assumed to be family (parents and grandparents are mentioned) but not explicitly stated.	Children aged under 13 years.	Qualitative: 6-8 female caregivers (in each of four FGDs).<br>Quantitative: 47 children aged under 13.	5 LTFU (not seen in the clinic for 3 months after picking up their last one-month supply of ART), 3 children died and 3 children discontinued ART.<br>401 of 451 recorded appointments for the 47 children before and on ART were recorded. Missing data was due to incomplete records (no date of visit entered in the visit form, no appointment given).	Socioeconomic status<br>Stigma<br>Health system	No quantitative measurement: report from qualitative section.	Instrumental<br>Instrumental support consisted of supporting children to take ART and to help them adhere to clinic visits.	Socioeconomic status: difficult to afford trips to the clinic and the ART drugs themselves (at the time, ART in Malawi was not free). Caregivers reduced the dose they gave their child to make the drugs last longer. Living in a rural location made travel more expensive.<br>Stigma: not disclosing, which meant there was no support for children taking pills when the primary caregiver was not present.<br>Health system: sometimes there were no drugs available.	Scheduled appointments, and caregiver assessment of missing doses.<br>Twenty-two out of 36 children alive and on ART by the end of July 2004, were fully adherent according to caregivers' reports throughout the study period.	n/a
**Xu et al., 2017 (89)**	Thailand.	Cross-sectional survey and in-depth interviews.	Primary caregiver: parents, grandparents and other family members.	Adolescents (parenterally infected with HIV).	Qualitative: 6 caregivers, 6 adolescents.<br>Quantitative: 568 child-caregiver pairs from the TEEWA study.	n/a	Socioeconomic status<br>Education<br>Cultural and social norms	Education:<br>None/ Primary school / Secondary school +.<br><br>Financial situation:<br>Ok, good, very good, very difficult, difficult.	Instrumental<br>Instrumental support consisted of reminders to take medications and instructions on doing so.	The quantitative study found no association between ART adherence and socioeconomic status, while the qualitative study found financial concerns to negatively impact adherence.	1) adolescent's self-reported missed doses in the past 7 days; 2) caregiver's rating of overall adherence (very good, good, average, poor, very poor).	Laboratory records of latest viral load.
